# Extracellular vesicle formation in *Euryarchaeota* is driven by a small GTPase

**DOI:** 10.1073/pnas.2311321121

**Published:** 2024-02-26

**Authors:** Joshua Mills, L. Johanna Gebhard, Florence Schubotz, Anna Shevchenko, Daan R. Speth, Yan Liao, Iain G. Duggin, Anita Marchfelder, Susanne Erdmann

**Affiliations:** ^a^Archaeal Virology, Max Planck Institute for Marine Microbiology, Bremen 28359, Germany; ^b^MARUM Center for Marine Environmental Sciences, University of Bremen, Bremen 28359, Germany; ^c^Max Planck Institute of Molecular Cell Biology and Genetics, Dresden 01307, Germany; ^d^Department of Biogeochemistry, Max Planck Institute for Marine Microbiology, Bremen 28359, Germany; ^e^The Australian Institute for Microbiology and Infection, University of Technology Sydney, Sydney, NSW 2007, Australia; ^f^Biology II, Ulm University, Ulm 89081, Germany

**Keywords:** extracellular vesicles, Archaea, small RNAs, small GTPase, *Haloferax volcanii*

## Abstract

Since their discovery, extracellular vesicles (EVs) have changed our view on how organisms interact with their extracellular world. EVs are able to traffic a diverse array of molecules across different species and even domains, facilitating numerous functions. In this study, we investigate EV production in *Euryarchaeota,* using the model organism *Haloferax volcanii*. We uncover that EVs enclose RNA, with specific transcripts preferentially enriched, including those with regulatory potential, and conclude that EVs can act as an RNA communication system between haloarchaea. We demonstrate the key role of an EV-associated small GTPase for EV formation in *H. volcanii* that is also present across other diverse evolutionary branches of Archaea. We propose the name, ArvA, for the identified family of archaeal vesiculating GTPases. Additionally, we show that two genes in the same operon with *arvA* (*arvB* and *arvC*) are also involved in EV formation. Both, *arvB* and *arvC*, are closely associated with *arvA* in the majority of other archaea encoding ArvA. Our work demonstrates that small GTPases involved in membrane deformation and vesiculation, ubiquitous in Eukaryotes, are also present in Archaea and are widely distributed across diverse archaeal phyla.

## Significance

Extracellular vesicles (EVs) play important roles in intercellular communication by transferring proteins, nucleic acids, lipids, and metabolites between cells. Few studies have investigated their role in Archaea. Here, we show that EVs of halophilic Archaea (haloarchaea), members of the *Euryarchaeota*, transfer an RNA cargo enriched in noncoding RNAs (ncRNAs), likely contributing to intercellular communication. We show that EV formation in haloarchaea is driven by a small guanosine triphosphatase (GTPase), ArvA, that is also conserved across other archaeal lineages, along with two genes closely associated with *arvA* that are also involved in vesicle production. Our work provides important insights into small GTPase-driven vesicle formation and a basis for further studies into the evolutionary relationships between prokaryotic and eukaryotic vesicle formation.

Extracellular vesicles (EVs) are small membrane-bound structures that bud off from the cellular envelope and are produced by living cells across all domains of life (1–3). They are able to enclose a wide range of cargo, including proteins, nucleic acids, and signaling molecules, facilitating a mechanism of interaction with the extracellular world. Communication mediated through EVs provides specific advantages for the cell, such as protection of the cargo from environmental stressors and degradation, the concentration of specific molecules into a self-contained structure, and the potential for selective delivery to designated targets (4, 5). With the diversity of EV composition and the advantages of EV-based communication, prokaryotic EV trafficking has been connected to a wide range of cellular functions. EVs have been discovered to act as defense against viral infection and antibiotic stress (6), mediating Bacteria–host interactions through the trafficking of regulatory RNA (7, 8), and facilitating the transfer of genetic material between cells (9, 10). Both their ubiquity among organisms and cellular functions make EVs an exciting new field for exploring intercellular communication and expand our view of the dynamics driving microbial environments.

EVs are known to be present in marine and aquatic samples (11, 12), and they likely play important roles in regulating environmental microbial populations. However, while there is a considerable amount of research regarding the role of EVs in bacterial pathogenicity, fewer studies have investigated the role that EVs play in microbial ecology, and even less investigate EVs in Archaea. Within the archaeal domain, EVs from only a few organisms have been studied (13). For the *Thermoproteota* (formerly *Crenarchaeota*) genus, *Sulfolobus*, vesicles were found to enclose proteinaceous toxins (14) as well as fragmented genomic DNA (15). Members of the *Euryarchaeota* have also been found to produce EVs enriched with DNA such as *Thermococcus* (16)*. Halorubrum lacusprofundi* (17) was found to produce specialized EVs including plasmid-encoded proteins and plasmid DNA (named plasmid vesicles, PVs), and plasmid DNA was also found in EVs of *Thermococcus* species (18). These studies demonstrate the ability of archaeal EVs to transport DNA between cells, which suggests that EV production may play an important role in horizontal gene transfer in Archaea.

EV production in *Sulfolobus* has been linked to its cell division machinery that is driven by ESCRT (endosomal sorting complex required for transport)-like proteins (Cdv proteins) (15). The ESCRT system is well studied in Eukaryotes and is responsible for the sorting and production of exosomes and the budding of various viruses (19). However, ESCRT-like proteins are not present in most currently annotated *Euryarchaeota* genomes (20), suggesting that a different mechanism is responsible for EV production. Instead, proteins with homology to proteins of the eukaryotic intracellular vesicle trafficking system, such as a small GTPase (21) and potential components of a vesicle coat (22), were identified in both EVs and PVs from *Hrr. lacusprofundi*, a member of the *Euryarchaeota*, implying that multiple mechanisms of vesicle production exist within the archaeal domain (17). Intracellular vesicle trafficking in Eukaryotes is coordinated by different vesicle coat complexes, such as COPI, COPII, and clathrin coat complexes, each mediating the trafficking of cargo between different membrane-bound organelles (23). Vesicle formation is initiated by the activation of a small GTPase, allowing for the recruitment of the respective coat complex (22, 24). GTPase-mediated intracellular vesicle formation and ESCRT-mediated vesicle formation evolved from different pathways (25). Additionally, small GTPases and other proteins predicted to be related to components of the eukaryotic endomembrane system were found in the genomes of *Lokiarchaeota* and other Asgardarchaea (26–28), suggesting that the root of eukaryotic, small GTPase-dependent, intracellular vesicle formation lies within Archaea. Intracellular vesicle formation and membrane trafficking mechanisms are essential components of the eukaryotic endomembrane system and have been hypothesized to be crucial for the emergence of Eukaryotes (29). While there are hypotheses that argue for either bacterial or archaeal roots of the endomembrane system (27, 30, 31), the experimental evidence to support either hypothesis remains absent.

In order to understand EV production in *Euryarchaeota,* and in particular halophilic Archaea (haloarchaea), we used the model organism *Haloferax volcanii*, to investigate the composition of EVs as well as their capacity to transfer their cargo to other organisms. We observe particular RNAs being enriched in EVs of various haloarchaea and demonstrate that the RNA cargo can be transferred between cells of the same species. We also investigated the roles of various genes in EV production, including an EV-associated small GTPase, which suggests a mechanism for EV generation in halophilic Archaea that could be similar to intracellular vesicle trafficking in Eukaryotes. From our findings, we hypothesize that halophilic Archaea utilize EVs to communicate and potentially regulate the microbial community in hypersaline environments.

## Results

### EV Production in *H. volcanii* Is Dependent on Growth Conditions.

The production of EVs and a set of specific EV-associated proteins has been reported previously for the Haloarchaeon, *Hrr. lacusprofundi* (17). To investigate the generation and potential function of EVs in haloarchaea, we chose *H. volcanii* because it is a well-established model organism for haloarchaeal cell biology with a number of genetic tools available (32, 33). The capability of *H. volcanii* to produce EVs was also previously reported under UV irradiation (34).

**Fig. 1.**
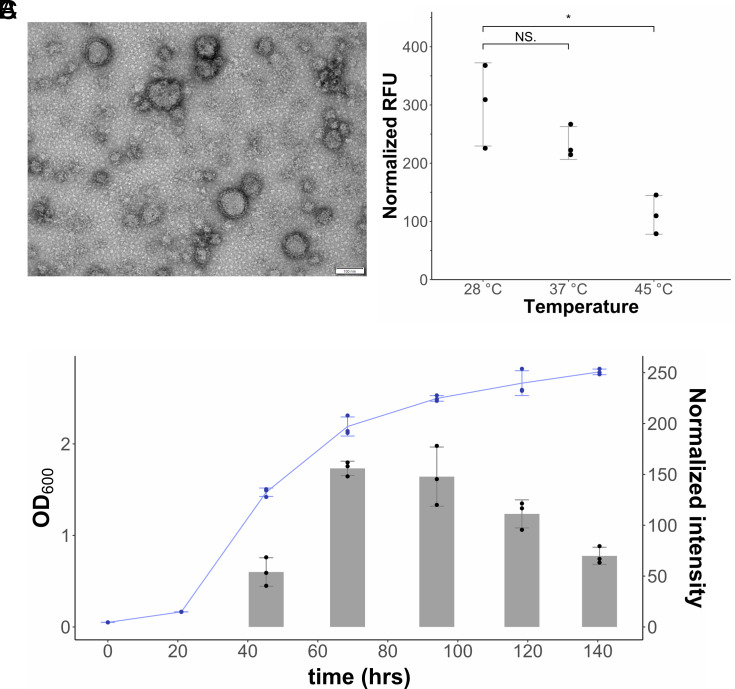
EV production in *H. volcanii DS2.* (*A*) Transmission electron micrograph of EVs. The size bar represents 100 nm. EVs were quantified from the supernatants of cultures (*B*) grown at different temperatures and (*C*) from different stages of growth at 28 °C. Each point indicates one biological replicate (n = 3). Error bars indicate the average of three biological replicates ± SD. Temperature-dependent EV production (*B*) was measured using relative fluorescence units (RFU) normalized to culture OD_600_. Significance is indicated above the graph (NS. indicates “not significant”, * indicates “*P* ≤ 0.05”). Growth-dependent EV production (*C*) was quantified by immunodetection measuring the intensity of signals on spot blot (original spot blot in *SI Appendix*, Fig. S2*C*), normalized to OD_600_. Growth of cultures indicated in blue follows *Left* axis, while EV production follows *Right* axis.

EVs were isolated from culture supernatants of *H. volcanii* and were observed to be spherical with a diameter ranging from 50 to 150 nm (Fig. 1*A*). Purification of EVs by iodixanol (OptiPrep™) density-based gradient purification resulted in EVs concentrating into two distinct bands in the gradient (*SI Appendix*, Fig. S1 *A* and *B*). No obvious differences distinguishing the two bands could be observed by TEM (*SI Appendix*, Fig. S1 *C* and *D*).

Initial efforts to isolate EVs close to the documented optimal temperature of *H. volcanii* at 45 °C yielded low amounts of EVs while lowering the temperature of growth to 28 °C increased EV yields, suggesting that EV production is temperature dependent. Therefore, we tested different growth temperatures using a fluorescence-based method for EV quantification. We observed a 2.7-fold decrease in EV production between 28 °C and 45 °C during the same stage of growth (*P* = 0.014) (Fig. 1*B*). EV production was determined to peak during the early stationary phase of growth (Fig. 1*C* and *SI Appendix*, Fig. S2*C*).

Since stress has also been reported to induce EV production, we tested environmental stress conditions such as UV exposure and virus infection using immunodetection-based EV quantification. UV stress induced a slight increase in EV production (1.3-fold increase, *P* = 0.025), while infection with the chronic infecting virus, HFPV-1 (35) slightly decreased EV production (1.27-fold decrease, *P* = 0.043) (*SI Appendix*, Fig. S2 *D* and *E*).

### *H. volcanii* EVs Are Associated with RNA.

**Fig. 2.**
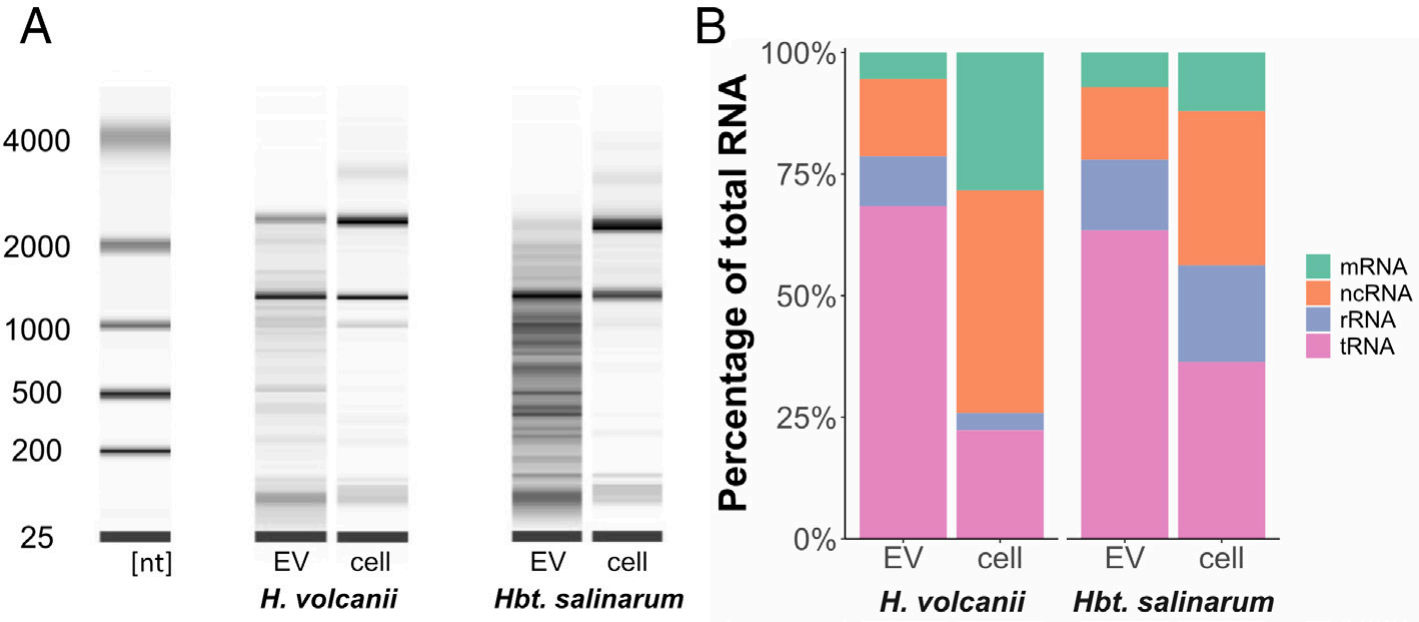
RNA composition of haloarchaeal EVs. (*A*) Analysis of the size distribution of RNA extracted from one replicate of purified EVs and whole cells of *H. volcanii* and *Hbt. salinarum*. (*B*) Expression levels of different RNA subpopulations calculated in percentage from total RNA expression (using TPM values) comparing cellular and EV-associated RNA for *H. volcanii* (average of three replicates) and *Hbt. salinarum* (one replicate).

EVs of both *Sulfolobus* (*Thermoproteota*) and *Thermococcus* (*Euryarchaeota*) were previously shown to enclose DNA (15, 16). To determine the nucleic acid contents of *H. volcanii* EVs, we attempted to isolate both DNA and RNA from a purified EV preparation. While DNA extraction yielded negligible amounts of DNA, RNA extraction revealed high yields of EV-associated RNA. Nuclease (DNase and RNase) treatment of the EVs prior to RNA extraction did not eliminate the presence of RNA, confirming that the transcripts are protected and likely enclosed within the vesicles. Analysis of the size distribution of the enclosed RNA revealed differences between EV-associated RNA and intracellular RNA (Fig. 2*A*). While ribosomal 16S and 23S rRNA subunits were prominent in both EV and cellular preparations, we observed populations of RNAs that are significantly enriched in EVs with a tendency toward smaller transcripts (*SI Appendix*, Fig. S3).

### EV–Associated RNA Is Enriched in tRNAs, rRNAs, and ncRNAs (Noncoding RNA).

Preliminary sequencing approaches of EV-associated RNA revealed that using small RNA libraries (enriching for transcripts below 150 nt in length) best reflects the RNA content of EVs (refer to *SI Appendix, Supplementary Results* for details). Additionally, we compared the RNA content of upper and lower EV bands in density gradients (*SI Appendix*, Table S4 and *Supplementary Results*), revealing that the RNA content alone is unlikely the major factor leading to two subpopulations of EVs. To determine RNA enriched in EVs, we normalized EV-associated with intracellular RNA levels at the time point of EV isolation (refer to *SI Appendix, Supplementary Results* for details).

**Fig. 3.**
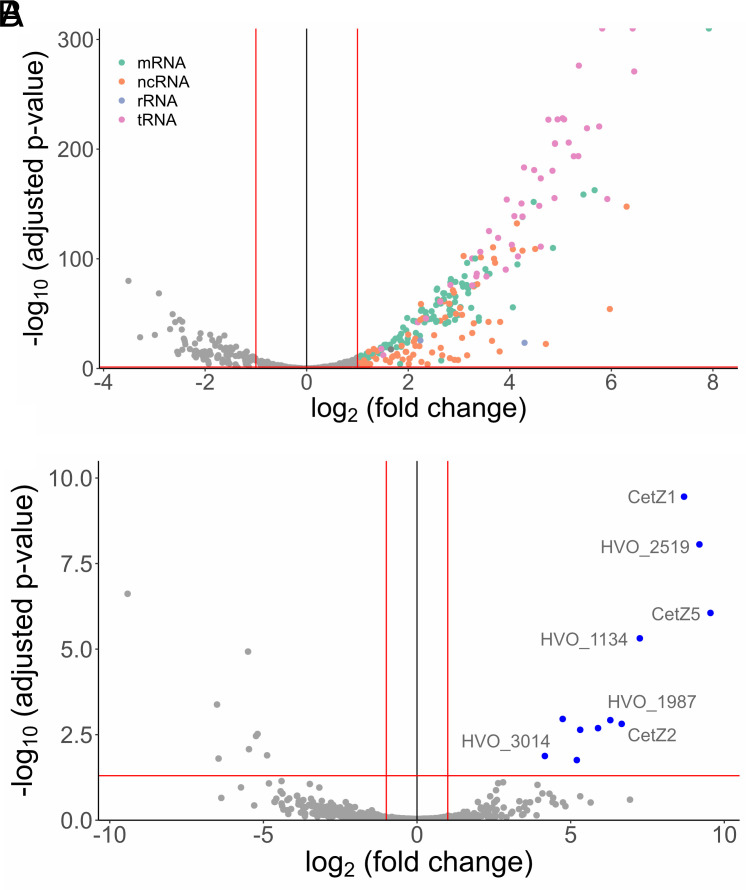
EV-associated RNA and proteins. Volcano plots of RNA (*A*) and protein (*B*) abundance in EVs in comparison to cellular RNA abundance and protein abundances from cell membranes. Differential RNA abundancies and adjusted *P*-values were calculated using DESeq2, and only transcripts with TPM > 10 are represented in this plot. Differential protein abundancies and adjusted *P*-values were calculated with DEP (*Methods*). Raw data are presented in *SI Appendix*, Tables S6 and S10. Red asymptotes indicate thresholds for enrichment (*P* = 0.05 and |fold change| = 2).

We identified around 4,400 genes represented by EV-associated transcripts, comprising the majority of the *H. volcanii* genome with around 79.5 ± 10.5% of the genome covered by at least one read (85.2 ± 0.8% for intracellular reads). Though this encompasses nearly all genes in the *H. volcanii* genome, only 474 of the transcripts identified had a TPM (transcript per million) greater than 10, suggesting that the majority of identified EV-associated RNA can be considered transcriptional noise. The most abundant of the identified transcripts were tRNAs (68.9 ± 2.1%), followed by ncRNA (transcripts that do not encode a protein, excluding rRNA and tRNA) and rRNAs (16S, 23S, and 5S) (16.1 ± 0.9% and 10.4 ± 1.2%, respectively) (Fig. 2*B*). The identified ncRNAs include intergenic sRNAs (36, 37) and antisense RNAs (asRNA). While we also detected mRNAs in the EV fraction, they only constitute about 4.6 ± 0.1% of the RNA population. Notably, when normalized to the intracellular RNA, the EV-associated RNA represented a unique subset of transcripts with little variation among replicates (Fig. 3*A* and *SI Appendix*, Table S6). We identified 230 transcripts as highly abundant (TPM > 10) and highly enriched (log2 > 1) in EVs. This population comprised tRNAs, rRNAs, ncRNAs, and mRNAs, with tRNAs being the most dominant group. Surprisingly, while the mRNA fraction was the least represented among EV-associated RNA, the most enriched (242-fold) among all transcripts was the mRNA for the S-layer glycoprotein (HVO_2072). A Northern blot analysis probing for the full-length mRNA (gene length 2,484 bp) in intracellular and EV-associated RNA revealed only small fragments of the transcript to be associated with EVs (*SI Appendix*, Fig. S4). While this could indicate that mRNAs are in general transferred as fragments in EVs, this has not been confirmed for each transcript. Besides HVO_2072 and mRNAs for various transposases, the remainder of highly enriched mRNAs were relatively low in abundance (TPM < 10).

Within the population of ncRNAs associated with EVs, we identified 74 ncRNAs that are both highly abundant and enriched in EVs (*SI Appendix*, Table S6). This population consists of intergenic RNAs as well as asRNAs. However, no function has been predicted for any of the intergenic ncRNAs so far. We also screened the ncRNAs for consensus sequences or a common secondary structure as specific selection markers for EV packaging; however, no common motif could be identified. Nevertheless, the identified asRNAs (21 asRNAs) appeared to exhibit sequence and structural similarities (*SI Appendix*, Fig. S5). The average length of these asRNAs was 45.5 nt (±5.8 nt), and all are associated with the 5′ end of ISH3-, ISH5-, ISH8-, ISH9-, and ISH11-type transposases from across the genome, overlapping with the predicted start codons of the respective transposase. This explained the abundance of highly enriched transposase mRNAs, which were actually the asRNAs.

While direct interactions between EVs and viruses have been documented (6, 38), we did not detect any changes to the transcriptional landscape of EVs derived from cultures infected with a chronic virus (*SI Appendix*, *Supplementary Results* and Fig. S6). However, we identified viral transcripts associated with EVs from infected cultures (*SI Appendix*, Fig. S7), suggesting that infected cells transport both host and virus-derived transcripts in EVs. However, it remains to be determined whether these transcripts are transferred as a whole or as fragments.

### Generation of RNA-Enriched EVs is Also Found among Other Haloarchaea.

EV production and presence of EV-associated RNA were tested in two other haloarchaeal organisms, *Halobacterium salinarum* and *Hrr. lacusprofundi*. EVs could be isolated from both organisms (*SI Appendix*, Fig. S8 *A* and *B*), and they were likewise found to be enriched in RNA. The size distribution of EV-associated RNA indicates an enrichment for a specific RNA population (Fig. 2*A* and *SI Appendix*, Fig. S8*C*).

RNA sequencing of *Hbt. salinarum* EVs revealed 85.4% of the *Hbt. salinarum* genome to be covered by at least one read from EV-associated RNA (94.5% from intracellular RNA library). The distribution of RNA populations was very similar between *H. volcanii* and *Hbt. salinarum* EVs (Fig. 2*B*), with the majority of EV-associated transcripts being tRNAs.

We identified 228 transcripts as highly abundant and highly enriched in *Hbt. salinarum* EVs (*SI Appendix*, Table S7). The transcript for the S-layer glycoprotein was also one of the most enriched EV-associated transcripts in *Hbt. salinarum*. The most enriched transcript was a 29 nt asRNA mapping to the coding region of VNG_RS00640, a predicted helix-turn-helix domain protein of unknown function. We also identified 16 highly enriched transposase-associated asRNA that associate with a larger range of transposase families than those from *H. volcanii*, some of which overlap with the respective predicted start codon. In total, 35 ncRNAs were identified as highly enriched and highly abundant in EVs of *Hbt. salinarum*. Of the ncRNA enriched in *Hbt. salinarum* EVs, 6 are sense-overlapping transposase-associated RNA (39), and 2 are intergenic sRNAs with high sequence identities to the predicted sRNAs from *H. volcanii*, HVO_2908s and H3.2 (36), that were also found in *H. volcanii* EVs.

### EVs Are Enriched with Specific Proteins.

The protein compositions of *H. volcanii* EVs and their respective cellular membranes were analyzed by mass spectrometry (LC–MS/MS). Comparison of upper and lower EV bands in gradients revealed no significant differences in protein content (*SI Appendix*, Fig. S9). Therefore, we concluded that protein content alone is most likely not the major factor causing the separation into two bands and pooled the results from both bands for further analysis.

In total, we identified 328 proteins associated with EVs and 668 proteins in the cellular membrane preparations. We compared the abundancies of proteins in EVs with those in cell membranes and obtained 11 proteins significantly enriched in EVs (log2 > 1, adjusted *P*-value < 0.05) (*SI Appendix*, Table S8 and Fig. 3*B*), including one protein exclusively detected in EVs (hypothetical protein, HVO_2519, with unknown function and no detectable conserved domains). Several CetZ proteins, including CetZ5 (HVO_2013), CetZ1 (HVO_2204), and CetZ2 (HVO_0745), were identified to be enriched in EVs. CetZ1 and CetZ2 have been shown to be involved in controlling cell shape and motility in *H. volcanii*, and the CetZ protein family has been predicted to be involved in other cell surface–related functions in Archaea (40, 41).

Other highly enriched proteins include FtsZ2 (cell division protein) (42), HVO_1134 (hypothetical protein), HVO_1987 (signal peptide peptidase SppA), HVO_2985 (hypothetical protein, no conserved domains), HVO_1964 (PRC-barrel domain), and HVO_B0079 (ABC transporter ATP-binding protein).

Most interesting was the enrichment of a small, single-domain GTPase, HVO_3014 (OapA) (Fig. 3*B*), an ortholog of the GTPase, Hlac_2746, which was also found to be enriched in *Hrr. lacusprofundi* EVs (17). OapA was initially thought to have an influence on genome replication due to its association with the origin of replication. However, despite a study characterizing a mutant strain, no distinct function could be assigned to OapA so far (43). Hidden Markov model (HMM)-based searches (44) identified similarities between the haloarchaeal, vesicle-associated GTPase, and other eukaryotic small GTPases involved in vesicle formation.

Differential expression analysis only identifies proteins that are present in higher abundancies in EVs than in cell membranes, leaving out other proteins that could be functionally relevant but are present in equal or lower abundancies when normalized to the cell membrane. For instance, the small GTPase, HVO_3014, was not identified to be enriched in EVs from UV-treated cells using a standard threshold (*SI Appendix*, *Supplementary Results*, Table S9, and Fig. S10), yet we observe its integral relationship to EV production in *H. volcanii* (see below). Therefore, we also identified the proteins that were found to be present among all 12 EV samples analyzed (*SI Appendix*, Table S10) and identified 285 proteins present across all samples. All proteins identified as enriched by differential expression analysis were also present in this list, except HVO_2399 identified as enriched only in EVs from UV-treated cells, suggesting that the protein composition slightly changes upon UV exposure. The most abundant protein was cytoskeletal protein, CetZ1 (HVO_2204), followed by the S-layer glycoprotein, HVO_2072. Other notable proteins within this list were ribonuclease J (HVO_2724), diadenylate cyclase (HVO_1660), and HEAT-PBS family protein (HVO_1020). RNase J is an exonuclease and could be relevant to the enrichment of RNAs found associated in the EVs. Diadenylate cyclases are responsible for the production of cyclic-di-AMP, a common secondary messenger among Bacteria and Archaea, including *H. volcanii* (45). HVO_ 1020 is a homolog (55% sequence identity) to *Hrr. lacusprofundi* Hlac_2402, which was also identified in *Hrr. lacusprofundi* EVs. HVO_1020 is predicted to contain an α-solenoid domain, which is found in adaptor proteins of eukaryotic intracellular vesicles coat complexes (25, 46).

### Knockout of the Small GTPase, OapA, Abolishes Formation of RNA-Associated EVs.

**Fig. 4.**
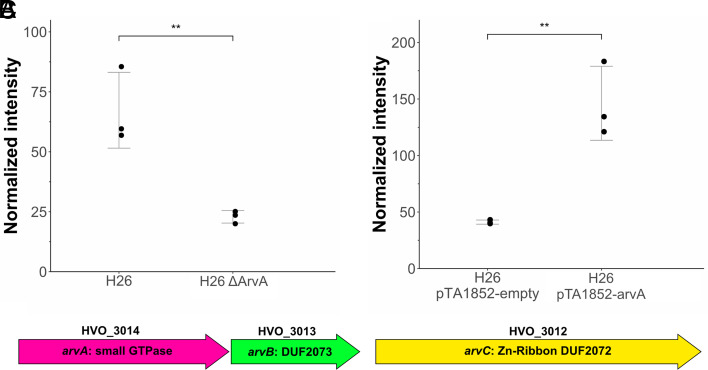
Analysis of *H. volcanii* EV-associated GTPase, ArvA. (*A*) Quantification of EVs in the culture supernatant of the ArvA knockout strain and the respective parental strain. (*B*) Quantification of EVs in the culture supernatant of a strain overexpressing ArvA (pTA1852-ArvA) compared to control empty vector (pTA1852-empty). EVs were quantified by immunodetection and were averaged over three replicates with error bars denoting one SD from the average value. Original spot blots are presented in *SI Appendix*, Fig. S11 *A* and *B*. (*C*) Map of the *ArvA* operon in *H. volcanii*.

To investigate the proposed involvement of OapA in EV production in *H. volcanii*, we compared the phenotypes of an OapA knockout strain (43) to the respective parental strain (H26). The OapA knockout strain yielded a dramatically reduced amount of EVs in comparison to the parental strain (about 2.94-fold reduction, *P*-value = 0.0086) (Fig. 4*A* and *SI Appendix*, Fig. S11*A*).

Gradient purification of concentrated OapA mutant supernatant resulted in either no distinct band or only one band with reduced intensity in density gradients of the parental strain (*SI Appendix*, Fig. S12 *A* and *B*). RNA extracted from this single band yielded very low RNA concentrations and was not detectable on a fragment analyzer. Therefore, we propose that the remaining particles isolated from supernatants of OapA knockout strain cultures (*SI Appendix*, Fig. S12 *C* and *D*) are vesicles deriving from lysed cells. Additionally, they could represent virus particles from a provirus region that was described to produce virus particles previously (47), which was confirmed to be active in the OapA mutant by genome sequencing (PRJEB58368). We conclude that the strain is unable to produce EVs associated with RNA. Phenotypic changes of the cell morphology were also observed for the knockout strain (*SI Appendix*, Fig. S13 *A* and *B*). The formation of rod-shaped cells appears to be less frequent when *oapA* is deleted. Interestingly, the OapA mutant also showed a slightly increased growth rate when compared to the parental strain under the conditions tested (*SI Appendix*, Fig. S13*C*).

Further, overexpression of OapA in a wild-type background strain (H26) resulted in increased vesicle production (3.5-fold increase, *P*-value = 0.0051) (Fig. 4*B* and *SI Appendix*, Fig. S11*B*). The hypervesiculation phenotype could also be observed by TEM (*SI Appendix*, Fig. S11*D*), further implicating the crucial role of OapA for EV production in haloarchaea. Therefore, we propose the name, ArvA, for the newly identified archaeal vesiculating GTPase.

Two genes (HVO_3013, *oapB*, and HVO_3012, *oapC*) are located in the same operon with *oapA* (Fig. 4*C*), and we identified these genes to be associated with *oapA* homologs in other archaeal lineages (see paragraph “*Archaeal vesiculating GTPase, ArvA, is conserved among various archaeal clades*”). Therefore, we will refer to them as ArvB (OapB) and ArvC (OapC). ArvB contains a DUF2073 domain (unknown function) and ArvC a DUF2072 domain (Zn-Ribbon domain of unknown function). Analysis of the predicted tertiary structure [Alphafold2 (48)] of ArvC did not allow solid conclusions about the function. However, the predicted structure of ArvB showed structural similarity with the tertiary structure of SepF (3ZIG) (*SI Appendix*, Fig. S14). We also investigated their role for EV production in *H. volcanii*, despite the fact that none of the two proteins were identified as EV-associated proteins by MS. The knockout strain for ArvB resulted in a 3.13-fold reduction in EV production (*P* = 0.001), while the knockout strain for the Zn-ribbon protein ArvC resulted in a 1.65-fold increase in EV production (*P* = 0.02) (*SI Appendix*, Fig. S15).

### EV–Associated RNA Is taken up by *H. volcanii* Cells.

In order to test the ability for EVs to deliver the RNA cargo to a target organism, we used ^14^C-labeled uracil as a reporter to track the movement of RNA. EV preparations from the EV-defective ArvA knockout strain served as a control.

**Fig. 5.**
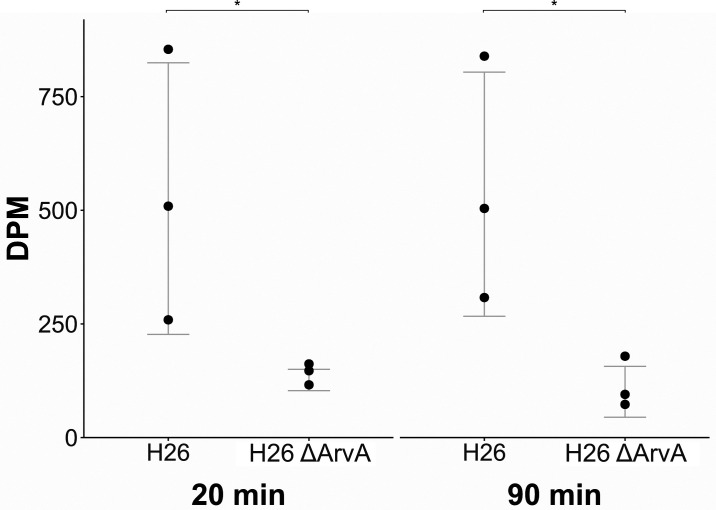
Transfer of radioactively labeled RNA by EVs. EVs were isolated from cells (H26 and H26 ΔArvA) that were incubated with radiolabeled uracil, resulting in EVs associated with radiolabeled RNA. EVs were then incubated with nonlabeled wild-type cells and the intracellular radioactivity in decays per minute (DPM) was measured 20 and 90 min postincubation, and normalized by subtracting background radiation (~15 DPM). Significance was calculated using a one-tailed *t* test (* indicates *P* < 0.05).

About 98% of the introduced radioactivity was taken up by both the parental strain and the ArvA knockout strain over 6 d of growth. Subsequently, 1.90% of the radioactivity was detected in EV preparations of the parental strain, whereas only 0.11% was detected in EV preparations of the ArvA knockout strain. After 20 min of incubation of the labeled EV preparations with fresh cells, we could detect a transfer of radioactivity into the unlabeled cells, with parental strain EVs transferring significantly more radioactivity than the ArvA knockout strain EV preparation (*P* = 0.04) (Fig. 5). Measurements after 90 min of incubation did not show a change of radioactive uptake from EVs of both strains (*P* = 0.025 between uptake from parental strain and ArvA knockout strain-prepared EVs), indicating that the transfer was already complete after 20 min. Thereby we confirm that the RNA enclosed in *H. volcanii* EVs can be taken up by *H. volcanii* cells in a short time frame. While we strongly assume that the EV-RNA is internalized by the receiving cells, we cannot exclude that we detect RNA containing EVs that are strongly bound to the cells and were not removed by washing.

### Archaeal Vesiculating GTPase, ArvA, Is Conserved among Various Archaeal Clades.

To get an overview of whether the archaeal vesiculating GTPase, ArvA, is present in other Archaea, we searched for proteins with high similarity to HVO_3014 against archaeal and bacterial GTDB (genome taxonomy database) species representatives using an alignment score ratio approach (*Methods*) (49). A total of 1,666 ArvA homologs were identified across 14 phyla of Archaea, with an uneven distribution of ArvA across these phyla (*SI Appendix*, Fig. S16 and Table S12). The majority of ArvA homologs were identified among the *Euryarchaeota* (*Halobacteriota* and *Methanobacteriota*), as well as in 7 DPANN phyla, including *Nanoarchaeota*, *Nanohaloarchaeota*, and *Altiarchaeota*. Interestingly, only 8 *Korarchaeota* out of 970 *Thermoproteota* genomes analyzed contained a homologous small GTPase. Further, only 8 out of 183 *Asgardarchaeota* genomes were identified to contain an ArvA homolog. Notably, we were also unable to identify any homologs in two well-studied EV-producing organisms: *Sulfolobus* (*Thermoproteota*), which is known to generate EVs using ESCRT-like proteins, and *Thermococcus* (*Methanobacteriota_B*), for which the mechanisms of EV formation has not been determined (15, 50).

**Fig. 6.**
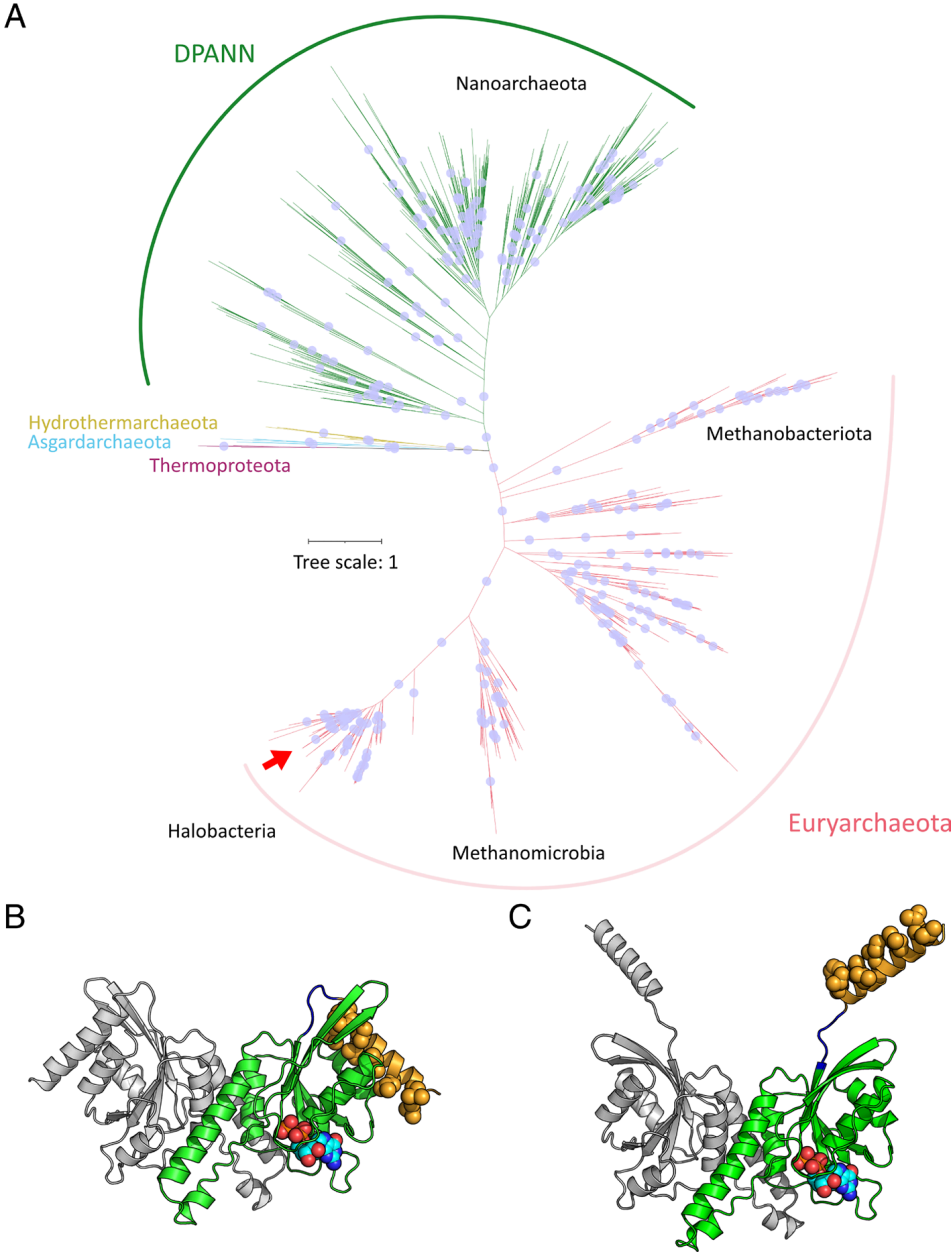
The new family of archaeal vesiculating GTPases, ArvA. (*A*) Unrooted phylogenetic tree of the identified ArvA homologs across the archaeal domain. The red arrow indicates position of *H. volcanii* ArvA. Blue dots represent branches with bootstrap value greater than 95. Structural prediction of tertiary structure of the ArvA dimer (monomer depicted in green) with (*B*) closed and (*C*) open conformations [AlphaFold v2 (41, 42)]. The modeled GDP ligand (displayed as balls), comes from the distant structural homolog EngA from *Thermus thermophilus* HB8 (rmsd of 3.29-Å out of 69 C-alphas, PDB 2DYK). Hydrophobic residues on N-terminal α-helix (displayed in yellow) are highlighted as balls.

A phylogenetic tree was constructed from the alignment of ArvA homologs (Fig. 6*A*). Both DPANN and *Euryarchaeota* form well-supported (≥99% bootstrap) distinct clades. *Asgardarchaeota*, *Thermoproteota*, and *Hydrothermarchaeota* form a third clade, however, not as well-supported (bootstrap value of 56). Overall, the ArvA phylogeny agrees well with the taxonomy of the respective organisms. While we cannot rule out horizontal transfer events during the emergence of ArvA, evidence suggests that ArvA diverged separately within Euryarchaota and DPANN and might have been inherited vertically. While ArvA is currently annotated as PF01926 (50S ribosome-binding GTPase), an HMM-based search (44, 51) of *H. volcanii* ArvA against the Pfam database (Pfam-A_v36) revealed PF00025 (ADP-ribosylation factor family, Arf family) as closest hit (probability: 99.75, e-value: 7.1e-16). However, a number of other Pfam entries for GTPases were also identified with a probability greater than 90%, indicating that an in-depth phylogenetic analysis of ArvA in relation to other GTPases will be necessary for an appropriate classification.

Alphafold2 (48, 52) models of *H. volcanii* ArvA were computed as a homodimer (*SI Appendix*, Fig. S17) because this conformation is known to be required for the activity of the eukaryotic vesiculating small GTPases Arf1(53). Two different structural conformations were predicted that resemble the conformational changes occurring in the activation of eukaryotic Arf1 (54). ArvA and Arf1 are structurally similar but differ in their dimerization interface (PDB 7WQY, aligns with a rmsd of 2.347-Å out of 588 atoms). Both proteins contain an N-terminal amphipathic α-helix with hydrophobic residues that are either tucked with the main body of the protein (Fig. 6*B*) or released away from the main body of the protein (Fig. 6*C*). Further, the release of the α-helix and the alignment of the hydrophobic residues suggest that this protein might interact with the cell membrane in this conformation.

The downstream genes of ArvA (Fig. 4*C*) were analyzed in all 1,666 organisms in which we identified ArvA (*SI Appendix*, Table S13). For about 95% of genomes containing ArvA, we could identify an ArvB homolog with 93% located directly downstream of ArvA, while ArvC was identified in 91% with 78% located up to 2 genes downstream.

## Discussion

While more evidence arises that EVs play important roles in mediating important cellular functions in Bacteria and Eukaryota, there is still a disproportionate lack of information about the function and cargo of EVs in Archaea (13). EV production has been previously reported in haloarchaea (17, 34), and here, we used the haloarchaeal model organism, *H. volcanii*, to investigate the nature of these EVs and the mechanisms of EV production.

EV production by *H. volcanii* appeared to be influenced by temperature and growth phase, with the highest yields below reported optimal growth temperatures and during exponential and early stationary growth phases. Interestingly, we detected a drop in EV production as the cultures entered the late stationary phase. We suggest this to be due to the cells increasing the rate of uptake or preserving energy for other processes during this stage of growth. Infection with the membrane-surrounded virus, HFPV-1, yielded slightly lower EV production, which we attribute to the increased resources required for virus particle production. While a previous study showed increased EV production under UV exposure at 45 °C (34), we observed a negligible influence of UV exposure under the conditions tested (28 °C), which might be due to the fact that EV production is already increased at 28 °C when compared to 45 °C. Analysis of the nucleic acid content of EVs produced by *H. volcanii*, as well as other haloarchaea, revealed that EVs are associated with RNA, as it has been described for some bacterial and eukaryotic EVs (55, 56), indicating that RNA-associated EVs are conserved among all three domains of life. *Thermococcus onnurineus* (*Euryarchaeota*) has previously been reported to produce EVs containing RNA (50); however, no characterization of EV-associated RNA was carried out for this organism. Treatment of EVs with nucleases did not eliminate the presence of EV-associated RNA; therefore, we infer that the RNA is internalized within EVs.

The RNA composition of *H. volcanii* EVs appears to reflect intracellular levels to a certain extent when tested under normal growth conditions and under infection with a virus. However, there is a distinct population of transcripts associated with EVs that does not correlate with the relative intracellular abundance but is instead more enriched within EVs. The majority of highly enriched transcripts encode for tRNAs and rRNA, and we suggest that they are enriched due to both their structural stability and their high intracellular abundance. Both tRNAs and rRNAs have been observed at high abundancies in vesicle-associated transcriptomics in bacterial EVs (8, 57) and could therefore be a commonality among EVs from prokaryotic organisms. Interestingly, the most enriched mRNA (coding for the S-layer glycoprotein) that we detected was shown to be nonspecifically fragmented in the EV-associated RNA fraction; however, the processing of other mRNA transcripts will need to be determined individually. Since we could not identify a common sequence or structural motif that would allow for a specific selection of particular RNAs to be enclosed into EVs, we suggest that the size, stability, or both are a defining factor for packaging. Additionally, the positioning of an mRNA close to the cell envelope, such as the mRNA of the S-layer glycoprotein, could play a role in determining the RNA population of EVs. The results we obtained from EVs of viral infected cultures (*SI Appendix, Supplementary Results*) showed that the RNA composition did not change significantly upon infection in both cells and EVs; however, we detected viral RNAs in the cells and subsequently also in EVs, clearly demonstrating that the RNA content of EVs represents the current transcriptional state of the EV-producing cell. When exposing cells to UV radiation, we subsequently observed changes to the RNA composition in EVs of UV-treated cells when compared to those of untreated cells (*SI Appendix, Supplementary Results*). Considering that UV exposure is known to influence the transcriptional landscape in *H. volcanii* cells (34), we assume that the changes observed in EVs are reflecting changes in the cell. In conclusion, we propose that RNA is taken up randomly into EVs, with transcripts that are highly enriched in the cell as well as transcripts that are translated at the cell envelope being preferably packaged. The respective cargo could be processed within EVs by RNases present in the vesicles, such as an RNase J that was detected by MS in EVs, leading to the degradation of mRNAs and a selection toward more stable RNAs (ncRNAs, tRNAs, and rRNAs). Alternatively, there could also be a preselection for small-sized RNAs for packaging into EVs. Both scenarios lead to an RNA cargo representing a transcriptomic snapshot of the cell with a particular enrichment in RNAs with a regulatory potential (ncRNAs and tRNAs), as we observe in *H. volcanii* EVs.

The expression of ncRNAs in *H. volcanii* has been observed to shift dramatically under different conditions (58), and we predict that the population of packaged ncRNAs also reflects this shift. There are some notable, studied examples showing EV-packaged ncRNAs regulating gene expression in a receiving organism, such as EV-associated ncRNAs of *Vibrio fischeri* (8) and *Pseudomonas aeruginosa* (7). We identify ncRNAs with regulatory potential associated with *H. volcanii* and *Hbt. salinarium* EVs. For example, we find a number of asRNAs overlapping with the start codon of various transposases that could potentially modulate transposase activity in a receiving organism. Unfortunately, the other identified ncRNAs are currently uncharacterized or do not have predicted functions. We have demonstrated that EVs of *H. volcanii* are able to transfer RNA between cells and that RNA-associated EVs are also produced by other haloarchaea. Therefore, we propose that halophilic archaea produce EVs as an intercellular communication mechanism to reflect the current intracellular state of the organism, and possibly influence gene expression in the receiving cell, allowing a timely response to environmental stimuli.

Proteomic analysis of EVs allowed us to draw conclusions about the mechanisms of the formation of EVs in haloarchaea. We identified an EV-associated small GTPase (OapA), of which a homolog was previously identified in *Hrr. lacusprofundi* EVs (17). Manipulation of OapA expression had strong effects on EV production. While the knockout of OapA resulted in an EV-defective strain, overexpression of OapA leads to hypervesiculation, demonstrating the key role of this protein in EV formation in *H. volcanii*. The only other known system where small GTPases are crucial for the production and trafficking of various vesicles is the eukaryotic endomembrane system (59). The production of these vesicles requires the activation of the small Arf-family GTPase in order to recruit the coat complex, resulting in deformation of the membrane and subsequent budding of the vesicle (60, 61). Deletion of this protein in Eukaryotes results in the elimination in the production of these intracellular vesicles (62), and we have observed a similar suppression when knocking out the small GTPase in *H. volcanii*, proving the existence of an archaeal vesiculating (ArvA) GTPase that regulates vesicle production in Archaea. Structural prediction of ArvA as a homodimer reveals similarities to eukaryotic Arf-family GTPases. Two different structures were predicted for ArvA with the N-terminal α-helix either tucked into the protein or released away from the main body of the protein, opening extra room in the GDP binding site. This suggests that upon activation and incorporation of GTP, an amphiphilic α-helix is able to protrude and likely interacts with the cell membrane, similar to what has been observed for Arf1 (53, 63). Therefore, we predict that vesicle generation in haloarchaea follows a mechanism similar to eukaryotic endomembrane vesicle formation, in that activated ArvA uses the N-terminal α-helix to interact with the cell membrane. Activation would then promote membrane deformation either from ArvA itself or through recruitment of additional proteins.

We identified other proteins that could also play a role in EV function, such as those with enzymatic functions or transport related proteins. Enzymatic activity was detected for EVs from the abundant marine cyanobacterium, *Prochlorococcus* (64), suggesting that EV-associated proteins can facilitate specific reactions extracellularly. CetZ proteins were found particularly prominent in EVs of *H. volcanii* and *Hrr. lacusprofundi* (17). However, EV production was not altered in knockout strains of the respective CetZ proteins (*SI Appendix, Supplementary Results*), suggesting that they do not play a significant role in EV formation in *H. volcanii*. CetZ proteins are known to be associated with the cell envelope (40), and we assume that this loose association could lead to enclosing of CetZ proteins during EV formation. Components of ABC transport systems, in particular also solute-binding proteins of ABC transporters, make up the overall majority of proteins associated with EVs of *H. volcanii* and were also detected in high abundancies in EVs and PVs of *Hrr. lacusprofundi* (17) as well as other characterized EVs (15). While this enrichment could be due to their high abundance in the cell envelope, the binding capacity of the EV-associated solute-binding proteins could also allow sequestration of rare nutrients that could be incorporated by the receiving cell (65). Alternatively, EVs could play a role in the removal of obsolete proteins from the cell envelope, such as components of ABC transporters, allowing the cell to refresh the composition of the envelope to better adapt to their environment. Furthermore, we identified a highly enriched diadenylate cyclase, an enzyme involved in the formation of cyclic di-AMP. These molecules are known secondary messengers in *H. volcanii* (45) and could be enriched with EVs, providing an additional mechanism of communication.

Analysis of the lipid composition of EVs in comparison to the lipid composition of whole cells and cell membranes revealed some unexpected differences (*SI Appendix*, *Supplementary Results* and *Supplementary Discussion*). We were able to detect the major bilayer forming lipids PG-AR, Me-PGP-AR, S-2G-AR, C-AR, 2G-AR, and cardiolipins, that were previously described for *H. volcanii* (66, 67) in all samples. However, the lipid composition of EVs differed significantly to that of cells and cell membranes when comparing the relative abundance patterns of different lipid groups. EVs were observed to be enriched with saturated lipids as has been observed in other bacterial EVs (12, 68), suggesting that membrane rigidity may play a role in EV production (11). EVs of the hyperthermophilic *Sulfolobus solfataricus* were also shown to contain the same lipid species as their respective producing cells with significant shifts in the ratio of particular lipid compounds (69), similar to what we observe in *H. volcanii*. Differences between the lipid composition of cells and EVs suggests a specific enrichment of particular lipid compounds in the EVs. The enrichment of specific lipids, proteins and RNA in EVs, as well as the temperature-dependency, all point toward an active mechanism for EV production.

Since the GTPase ArvA appears to be central to EV formation in *H. volcanii*, we searched for homologous proteins in public databases. While ArvA is absent from organisms that have been shown to exhibit an alternative mechanisms for EV formation (16), we identified ArvA GTPases across not only haloarchaea and *Euryarchaeota* but also within other major branches in the archaeal domain, such as the deep-branching lineage of DPANN Archaea. This suggests that the ArvA-driven mechanism of EV production is widespread among specific clades of Archaea, and we propose to classify these GTPases as ArvA-family GTPases. Phylogenetic analyses of the ArvA GTPases show that they group in accordance with their taxonomy, suggesting that they could have been inherited vertically. However, without experimental investigation, we do not know whether all ArvA-family GTPases are involved in EV production; though the presence of ArvA in DPANN and *Euryarchaeota* shows that vesiculating GTPases evolved much earlier in evolution than previously thought (27, 28). Small eukaryote-like GTPases have been identified previously in Archaea and Bacteria, some of which clustering closely with known eukaryotic Ras-like or Arf-like GTPases (27, 70). While it has been speculated that the origins of intracellular vesicle trafficking could stem from a bacterial endosymbiont (30), there is no evidence for any similar mechanism to the eukaryotic endomembrane system existing in the bacterial domain. Rather, the evidence presented in this paper suggests that the mechanisms for GTPase-dependent membrane deformation and vesicle production already existed in Archaea as is suggested by other hypotheses (27, 71). Additionally, while we only provide one example of an archaeal GTPase that functions in EV production, this does not exclude the possibility for other homologs to facilitate invagination processes. Further investigation into the function of archaeal GTPases in other lineages (such as in DPANN) as well as mechanistic studies are required in order to draw conclusions on the nature of this novel family of GTPases.

We identified two genes downstream of ArvA (*arvB* and *arvC*) that are also present in the majority of other organisms encoding for ArvA. While ArvB is most often located directly downstream of ArvA, the position of ArvC is slightly less conserved. Both genes were also observed to be involved in vesicle formation. ArvC exhibits a Zn-finger domain, which is known to be a crucial component of GTPase activating proteins (GAP) for Arf-like GTPases (72, 73). GAPs are negative regulators of GTPases required for transitioning the GTPase from the active form (membrane-bound) to the inactive form (membrane-free). Since knockout of ArvC leads to overvesiculation, possibly due to dysregulation of ArvA, we suggest that this conserved Zn-finger protein could represent a GAP. Knockout of ArvB leads to an EV-defective strain, suggesting that ArvB could be the corresponding guanine nucleotide exchange factor (GEF). GEFs are required for inducing the release of GDP from the GTPase, allowing for the association of GTP and subsequent activation of the GTPase (74). However, neither ArvB nor ArvC show any homology to the functional domains in the eukaryotic GAPs and GEFs. Instead, the predicted tertiary structure of ArvB aligns best with SepF (rmsd 2.9). SepF was shown to be involved in cell division in Archaea by interacting with FtsZ, which also contains a GTPase domain (75, 76). SepF anchors FtsZ to the membrane through an N-terminal membrane-binding domain (77). However, this membrane-binding domain is not predicted in ArvB, making it difficult to conclude any functional similarities between ArvB and SepF. Therefore, experimental investigation is required to identify the role of both proteins (ArvB/C) in EV production in Archaea.

In summary, we show that EV production and the enclosing of RNA into EVs is common for multiple haloarchaeal species. We propose that the formation of EVs in haloarchaea is an active and conserved process, considering the conditionality of EV production along with their molecular composition that differs significantly from the originating cell, as well as the crucial involvement of a GTPase that is conserved among haloarchaea and other archaeal lineages. The enrichment of RNA with regulatory potential in EVs and the conservation of this process among different species lets us propose that halophilic Archaea utilize EVs as a communication mechanism, influencing gene expression at a population-wide scale, as it has been proposed for some Bacteria (7, 8). Our work suggests that vesiculating GTPases driving intracellular vesicle trafficking in Eukaryotes could have emerged from an archaeal ancestor, as it has been proposed earlier (17), and evolved earlier in evolution than previously thought. While both, an archaeal and a bacterial origin has been proposed (17, 27, 30), the experimental evidence presented in this work supports the hypothesis of an archaeal origin of the eukaryotic endomembrane system.

## Methods

### Strains and Media.

*H. volcanii* strains and other haloarchaea used in this study are summarized in *SI Appendix*, Table S1. *H. volcanii* was either grown in Hv-Ca supplemented with the SL10 trace elements and vitamins as described for DBCM2 (78) (Hv-Cab) or Hv-YPC supplemented with the same trace elements and vitamins (78). For auxotrophic strains, media were supplemented with uracil (50 µg/mL) and tryptophan (50 µg/mL), as required (*SI Appendix*, Table S1). *Hbt. salinarum* was grown as described in ref. 79. *Hrr. lacusprofundi* was grown in DBCM2 medium (78). UV treatment (0.05 J) was performed in a petri dish using a UV cross-linker (Biometra™). Infection of *H. volcanii* cultures with the virus HFPV-1 was performed as described in ref. 35. Cultures were grown in glass flasks aerobically at 120 rpm at the temperatures indicated.

### Generation of Knockout Strains.

To construct plasmids for the deletion of the *aglB* gene, PCR fragments of the upstream and downstream flanking sequences (~530 bp) (primers listed in *SI Appendix*, Table S2) were joined by Gibson assembly and ligated into pTA131 (80) using *Bam*HI and *Hind*III restriction sites. The resulting plasmid was demethylated (81) and transformed into *H. volcanii* H26 using the two-step procedure (pop-in and pop-out) (80). The oapA deletion strain (43) was obtained from Jörg Soppa and confirmed by genome sequencing. Library preparation (FS DNA Library, NEBNext® Ultra™) and sequencing (Illumina HiSeq3000, 2 × 150 bp, 1 Gigabase per sample) were performed at the Max Planck-Genome-Centre Cologne (Cologne, Germany).

### Isolation and Purification of EVs.

EVs from *H. volcanii* were isolated and purified as described in ref. 82 (for details, see *SI Appendix, Supplementary Methods*). Purification using an OptiPrep™ density gradient yielded two bands containing EVs.

EVs from *Hrr. lacusprofundi* were isolated and purified following methods in ref. 17. EVs from *Hbt. salinarum* were isolated as described for *H. volcanii*¸ with a growth temperature of 45 °C.

### Transmission Electron Microscopy.

Samples were adsorbed onto a carbon-coated copper grid (FCF200-Cu) for 3 min and negatively stained with 2% uranyl acetate for 45 s. Grids were imaged at 200 kV with JEOL JEM-2100 Plus transmission electron microscope.

### EV Quantification.

Two different quantification methods were used because each of them proved unsuitable for some conditions tested (see *SI Appendix, Supplementary Methods* for details).

EVs were quantified from 2 mL of culture supernatant after removal of cells through centrifugation at room temperature (~20,000 × g, 10 min twice, followed by 30 min) and filtration through a 0.22-µm pore filter.

For quantification using fluorescence labeling, MitoTracker® Green (Invitrogen) (final concentration 500 nM) was added to the EV solution containing 10% PEG6000, inverted to mix, and incubated at room temperature for 30 min. EVs were pelleted by centrifugation (~20,000 × g, 40 min, 4 °C). The EV pellet was then resuspended in 200 µL 22% buffered sea water (BSW) (78). Fluorescence was measured on a Spectrophotometer (DeNovix, DS-11 FX+) with blue excitation (470 nm) and emission between 514 to 567 nm. Background fluorescence was determined by performing the same procedure on sterile media. Normalized relative fluorescence units were determined by subtracting background fluorescence from each measurement and dividing by the OD_600_ of the respective culture at the time of harvesting.

For quantification using immunodetection, EVs were pelleted by centrifugation (20,000 × g, 4 °C, 40 min) after PEG precipitation (10% final concentration). The pellet was resuspended in 100 µL of 50 mM Tris-HCl to lyse EVs. Ten microliters of the EV preparation was spotted onto a nitrocellulose membrane (Bio-Rad) and dried for 1.5 h. Blocking was performed with blocking solution (60 g skimmed milk powder in 20 mL 1× TBS buffer [10× TBS buffer: 24 g/L Tris-HCl, 5.6 g/L Tris, and 88 g/L NaCl, with pH adjusted to pH 7.6 with HCl]) for 30 min, followed by incubation with the primary antibody [against HVO_2204, CetZ1 (40), that was found to be highly enriched in EVs (17)] 1:1,000 diluted in blocking solution for 1 h. The membrane was washed twice with 1× TBS-TT (10× TBS-TT is 10× TBS buffer with 5 mL/L Tween 20 and 5 mL/L Triton X) and once with 1× TBS before incubation with the secondary antibody (IgG anti-rabbit HRP conjugate, Promega) 1:1,000 diluted in blocking solution for 1 h. Washing steps were repeated and chemiluminescence was visualized using Clarity Western ECL Substrate (Bio-Rad). Chemiluminescence intensity was calculated using ImageJ (83).

### RNA Extraction and Transcriptomic analysis.

RNA was extracted from cell pellets or EV pellets using TRIzol™ (Thermo Fischer Scientific) (84) (see *SI Appendix, Supplementary Methods* for details). Total RNA libraries (NEBNext® Ultra™ II RNA Library Prep Kit for Illumina), and small RNA libraries (RealSeq®-AC miRNA Library Kit) were prepared and sequenced (1× 150 bp, 1 Gb per sample) at the Max Planck-Genome-Center (Cologne, Germany). Preliminary RNA sequencing experiments (*SI Appendix, Supplementary Results*) were conducted with one replicate, while the final RNA sequencing for both untreated and HFPV-1 infected *H. volcanii* were performed in triplicates. RNA sequencing for *Hbt. salinarum* was conducted with one replicate of cellular RNA, and two replicates of EV-associated RNA that were pooled together after sequencing. Read mapping and calculations of gene expression and differential expression were performed using Geneious Prime® (2021.0.1). Reads were mapped to a compiled version of all genomic elements using the Geneious mapper (including a standard read trimming step) with 99% minimum overlap identity (90% minimum overlap identity for preliminary *H. volcanii* read mapping and *Hbt. salinarum* RNAseq). For samples with 3 or more replicates, differential expression was calculated with DESeq2, thereby normalizing EV-associated RNA to intracellular RNA. For samples with only one replicate, the default Geneious differential expression calculator was used. Transcripts were considered significant if transcripts per million (TPM) was greater than 10, log2 fold change was greater than 1, and *P*-value was lower than 0.05. Consensus sequences were predicted using MEME (85) with default settings. Sequence alignment and structural alignment among asRNAs were predicted using LocARNA (86–88), with the temperature setting set to 28 °C.

### Northern Blot.

The Northern blotting protocol was adapted from ref. 89 (see *SI Appendix, Supplementary Methods* for details).

### Plasmid Construction and Expression of OapA.

The coding region for *oapA* was amplified by PCR (primers listed in *SI Appendix*, Table S2) and ligated into pTA1852 (see *SI Appendix, Supplementary Methods* for details) using *Pci*I and *Eco*RI restriction sites. The resulting plasmid was demethylated (81) and transformed into *H. volcanii* strain H26 (90).

Expression of tagged OapA (OapA_t_) was adapted from ref. 91. Transformed strains were grown in Hv-YPC supplemented with 200 µg/mL tryptophan at 28 °C until OD_600_ of approximately 1. Cultures were then supplemented with 18% BSW containing 5 mg/mL tryptophan (final concentration of 450 µg/mL tryptophan). Cultures were grown for 2 h at 28 °C before EVs were quantified as described. Affinity purification of OapA_t_ was modified from ref. 92 (see *SI Appendix, Supplementary Methods* for details).

### Protein Extraction and Analysis.

Protein content of EVs was compared with protein content of cell membranes as described previously (17). Proteins were isolated from purified EVs (triplicates of each upper band and lower band in density gradients) and host membranes (in triplicates) from untreated and UV-treated samples as described in ref. 82. TCA precipitated proteins were dissolved in 30 µL 1 × Laemmli sample buffer and separated (3 cm) on Any kD™ Mini-PROTEAN® TGX™ Precast Protein Gels (Bio-Rad Laboratories, Germany). The gels were visualized with Coomassie staining and each gel lanes cut into two slabs, which were processed individually. Proteins were in-gel reduced with dithiothreitol, alkylated with iodoacetamide, and digested overnight with trypsin (Promega Mannheim, Germany). Resulting peptide mixtures were extracted twice by exchange of 5% of formic acid (FA) and acetonitrile, extracts pooled together and dried down in a vacuum centrifuge. Peptides were then resuspended in 25 µL of 5% FA, and a 5-µL aliquot was analyzed by LC–MS/MS on a nano-UPLC system Ultimate3000 series interfaced to a LTQ Orbitrap-Velos mass spectrometer (both Thermo Fisher Scientific, Bremen, Germany). The nano-UPLC was equipped with an Acclaim PepMap100 C18 75 µm i.d. × 20 mm trap column and a 75 µm × 15 cm analytical column (3 µm/100 Å, Thermo Fisher Scientific, Bremen, Germany). Peptides were separated using 80 min linear gradient; solvent A was 0.1% aqueous FA and solvent B was 0.1% FA in neat acetonitrile. Spectra were acquired using Data Dependent Acquisition method and Top 20 approach; lock mass was set on *m/z* = 445.1200 (polydimethylcyclosiloxane). Three blank runs were performed after each sample analysis to avoid carryover. Acquired spectra were searched against *H. volcanii* proteins in the NCBI database (June 2020, 12,045 entries) by MaxQuant software (v. 1.6.10.43) using default settings and matched between runs option. False discovery rate was 1% and variable modifications were methionine oxidized, cysteine carbamidomethylated and propionamide. Two miscleavages were allowed and minimal number of matched peptides was set to two. Relative quantification was performed using LFQ intensity values calculated by MaxQuant. Proteins were only considered present in EVs if matched with two or more peptides in all EV samples for that condition, and a respective LFQ value was identified in all EV samples for that condition.

Differential expression of proteins was calculated using R package, DEP (differential enrichment analysis of proteomics data) (v. 1.21.0) (93), based on the LFQ intensity values generated by MaxQuant. We analyzed biological triplicates of upper and lower EV bands in OptiPrep™ gradients separately for protein content. However, only 1 protein was found to be more abundant in the upper band and 2 more abundant in the lower band, while the majority of proteins appeared to be consistent between upper and lower bands (*SI Appendix*, Fig. S9). Therefore, results from upper and lower bands were pooled for a total of 6 biological replicates of EV samples and compared to 3 biological replicates of cell membrane samples. The threshold for significant enrichment in EVs was a log2 fold change greater than 1 and adjusted *P*-value lower than 0.05.

### Identification and Phylogenetic Analysis of small GTPases and Associated Proteins across the Archaeal Domain.

Tertiary structure of the OapA dimer was predicted with AlphaFold v2 (48, 52). Homologs of small GTPase, HVO_3014, were identified in major archaeal clades using DELTA-BLAST (default settings), and only hits that contained a complete GTPase binding domain [as determined with Interpro (94)] and had a similar length (<250 aa) were included. This resulted in 21 sequences from different organisms, including *H. volcanii* and *Hrr. lacusprofundi*. These 21 sequences were used as a reference database in a DIAMOND (95) search (score cutoff = 50), with the query consiting of the entire protein content of a nonredundant set of 78,768 archaeal and bacterial genomes. The query was generated from the GTDB species representatives (r207) (96) and the global catalog of earth’s microbiomes OTU dataset (97) dereplicated at 95% average nucleotide identity using fastANI (98). This search resulted in 96,121 hits, and 1,686 true positive hits were subsequently selected using an alignment score ratio approach, allowing us to identify sequences with both high identity to the proteins of interest as well as a similar length (49, 99). This set was further manually curated, removing the only five bacterial GTPases based on protein phylogeny using FastTree 2 (100) and MUSCLE (101), as well as removing 15 sequences longer than 250 amino acids. This resulted in a final protein set of 1,666 archaeal GTPase sequences. The final dataset was aligned with MUSCLE and a phylogenetic tree was constructed using IQ-Tree (102) with ultrafast bootstrap analysis (103) using 1,000 bootstrap replicates and default settings, auto-selecting the substitution model (104). The phylogenetic tree was visualized on iTOL (v 6.6) (105) as unrooted, and taxonomy was mapped onto the resulting tree. The same approach was used to identify ArvB and ArvC homologs (for details refer to *SI Appendix, Supplementary Methods*).

### Tracking of EV Uptake Using 2-14C Uracil.

To generate EVs containing radiolabeled RNA, uracil auxotrophic parental strain, H26 (80), and uracil auxotrophic deletion mutant H26Δ*oapA* were inoculated with an optical density (600 nm) of 0.05 into 50 mL of Hv-Cab supplemented with a mix of unlabeled uracil and 14C-labeled uracil (8.621 µg/mL final concentration, 25 µCi per culture), each in triplicates. Cultures were grown at 28 °C for 7 d before EVs were harvested. To harvest the EVs, cells were pelleted by centrifugation (4,000 × g, 1 h). The supernatant was filtered through a 0.22 µm pore filter to remove the remainder of larger contaminants. EVs in the flow-through were then concentrated with Vivaspin® 20 (10,000 MWCO, Sartorius). The filters were washed three times with 22% BSW to remove residual unincorporated 14C uracil and subsequently concentrated to 500 µL of radiolabeled EVs per replicate. *H. volcanii DS2* was grown in Hv-YPC media at 45 °C until OD (600 nm) of 1. 60 mL of culture were harvested by centrifugation (4,000 × g, 20 min), washed with 6 mL HV-YPC and subsequently resuspended in 6 mL Hv-YPC. For each replicate, 500 µL of cell concentrate were incubated with the 500 µL of EV concentrate in a heat block at 28 °C, 300 rpm. After 20 and 90 min postincubation, 300 µL were removed for measurement. The cells were pelleted (5 min, 10,000 × g) and washed 3 times with 22% BSW to remove any residual EVs present. The resulting cell pellet was resuspended in 500 µL 22% BSW, added to 4 mL scintillation fluid (Ultima Gold™ XR, Perkin Elmer), and measured in a scintillation counter (Tri-Carb 4910 TR, Perkin Elmer). Significance was calculated using the one-tailed *t* test.

## Supplementary Material

Appendix 01 (PDF)

Dataset S01 (XLSX)

Dataset S02 (XLSX)

Dataset S03 (XLSX)

Dataset S04 (XLSX)

Dataset S05 (XLSX)

Dataset S06 (XLSX)

Dataset S07 (XLSX)

Dataset S08 (XLSX)

Dataset S09 (XLSX)

Dataset S10 (XLSX)

Dataset S11 (XLSX)

## Data Availability

Raw data for resequencing of H26 Δ*oapA* mutant are available at ENA under project number PRJEB58368. Raw data for all RNA sequencing experiments for *H. volcanii* and *Hbt. salinarum* are available at ENA under project numbers PRJEB58342 and PRJEB58367, respectively. The MS proteomics data have been deposited to the ProteomeXchange Consortium via the PRIDE (106) partner repository with the dataset identifier PXD038319 and 10.6019/PXD038319.
