# Supplementary material for: Extracellular vesicle formation in Euryarchaeota is driven by a small GTPase
Source: Proc Natl Acad Sci U S A. 2024 Feb 26;121(10):e2311321121. doi: 10.1073/pnas.2311321121 (PMC10927574; doi:10.1073/pnas.2311321121)
Supplement: Supplementary file 1 — Appendix 01 (PDF) [file pnas.2311321121.sapp.pdf]

SUPPLEMENTAL MATERIAL

Supplementary Results

EV-associated RNA is best analyzed when using small RNA libraries and normalizing EV RNA content with host cell RNA content

To determine the nature of the EV enclosed RNA, total RNA and small RNA (enriching for transcripts below 150 nt in length) libraries were prepared from EV-extracted RNA. When comparing sequencing results from both libraries, we observed a drastically different transcriptional profile (Supplementary Table 3). Around 95% of reads from total RNAseq mapped to ribosomal RNA (rRNA) and only 17 transcripts recruited enough reads to reach the threshold (TPM > 10). In contrast, the small RNA library revealed a more diverse array of transcripts with transfer RNA (tRNA) being the most dominant RNA species (around 85% of reads) and 264 transcripts identified within the threshold. Further, over 2000 transcripts were only identified in the small RNA library and not in the total RNA library, the majority of them being tRNAs and non-coding RNAs (ncRNA), indicating that the total RNA library excludes important smaller transcripts. Therefore, we decided to use small RNA libraries for further analysis of EV-associated RNAs, as this seems to yield a more accurate picture of the RNA composition of EVs.

We also compared transcripts from EVs in the upper and lower bands in density gradients to determine whether the bands represented different subpopulations of EVs with respect to RNA content (Supplementary Table 4). Indeed, we identified transcripts that were only present in the upper band (app. 200) or only in the lower band (80). However, the abundance of these transcripts was below the threshold (TPM > 10) and they were disregarded. Overall, the RNA composition between both bands was mostly identical, with few outliers. We concluded that the RNA composition alone is not the differentiating factor between the two subpopulations of EVs in different density gradient bands, and pooling the bands for further sequencing analysis is acceptable.

Since vesicle production could be linked to UV exposure [1], we aimed to determine whether subjecting cultures to UV radiation would alter the RNA composition of EVs. Indeed, 145 transcripts appeared to be present in a higher abundance (log2 fold change > 1) in the UV-treated sample, and 32 transcripts were present in a higher abundance (log 2 fold change < -1) in the untreated sample (Supplementary Table 5). This population of EV-associated RNA from UV-treated cultures included all forms of RNA, including mRNAs, tRNAs, rRNAs and ncRNAs. Nevertheless, we realized that without determining transcriptional changes within the cells, it is difficult to distinguish transcripts that are associated with EVs as a response to UV exposure and transcripts that are present in EVs simply due to changes in intracellular levels. In order to differentiate between random packaging and potentially selective packaging of RNA into EVs, it became

clear that sequencing intracellular RNA at the time of EV harvesting was imperative for any kind of analysis.

Analysis of EV-associated RNA under infection with a virus reveals viral transcripts associated with EVs

Direct interactions between EVs and viruses have been documented, demonstrating the capacity for EVs to act as a viral defense mechanism [2] or to facilitate viral propagation [3]. While we detected only minor changes to EV quantities under infection with the virus HFPV-1 (Figure 2B), we wanted to test whether infection with HFPV-1 would influence the RNA composition of EVs and thereby possibly indirectly influence virus-host interactions.

While it was shown that infection with HFPV-1 drastically altered the transcriptomic landscape of the cell during exponential and early stationary growth [4], sRNAseq in late exponential growth revealed a nearly identical transcriptional profile when comparing infected and uninfected cells (Supplementary Figure 6A). Only two genes showed a significant upregulation (log2 > 1) in the infected cells, HVO_2657 and HVO_0272; however, both are in general rather weakly expressed (TPM < 15). When comparing the RNA content of EVs between infected and uninfected cells, two transcripts were found to be significantly higher in abundance in EVs of infected cells: HVO_A0466 and HVO_0272 (Supplementary Figure 6A). While HVO_0272 mRNA was about 4-fold upregulated in infected cells (log2 ~ 2), it was about 10-fold upregulated (log2 ~ 5) in EVs of infected cells (Supplementary Figure 6B), indicating that the packaging of this transcript into EVs increases significantly upon infection. Surprisingly, it appeared that the majority of reads mapping to HVO_0272 only map to two short regions of about 30 nt within the coding region of the gene that are identical to a region on the viral genome. Therefore, we conclude that the upregulation of HVO_0272 is due to viral transcripts mapping to the host genome.

Subsequently, when mapping reads to the virus genome, we detected a significant amount of viral transcripts in EVs. While only 1.7 ± 0.07% of intracellular RNA mapped to the HFPV-1 genome, 4.0 ± 0.10% of EV-associated RNA mapped to the viral genome, suggesting a slight enrichment of virus-derived transcripts in EVs. Both cellular and EV-associated RNA mapped the entire HFPV-1 genome, and no enrichment of particular viral RNAs could be detected in EVs (Supplementary Figure 7). However, the detection of viral transcripts within EVs shows that they are also exported in EVs together with host RNA.

Analysis of proteins in EVs from UV-treated cultures did not reveal significant differences

We also analyzed the protein composition of EVs from UV-treated cells and compared them with membrane-associated proteins isolated from their respective cells, to determine whether UV treatment would alter protein composition of the EVs. We identified 377 proteins associated with EVs and 668

proteins associated with their respective cell membranes. We identified 11 proteins to be enriched in EVs from UV-treated cells (Supplementary Table 9, Supplementary Figure 10A). All proteins identified as enriched in EVs from untreated cells were also identified as enriched in EVs from UV-treated cells, except for the small GTPase, HVO_3014, that was calculated as equally enriched but did not pass the p-value threshold. Instead, one additional ABC transport protein (HVO_2399) was identified as enriched. In comparing EV-associated proteins between untreated and UV-treated cultures, we did not identify major differences (Supplementary Figure 10B). Only one protein (HVO_B0027) was identified to be more enriched in EVs from untreated cultures, while two proteins (HVO_1751 and HVO_2529) were identified to be more enriched in EVs from UV-treated cultures. None of the enriched proteins from either preparation held functions that appear significant to EV production.

Testing other knockout mutants provides further insight into the mechanisms of EV formation

CetZ1 and CetZ2 were amongst the most abundant proteins in EVs; however, we were able to isolate EVs from the supernatant of both CetZ1 and CetZ2 knockout strains (Supplementary Figure 18). While quantification of EVs by the immunodetection-based method was not possible for the CetZ1 knockout strain, we did not have any indication when purifying EVs that EV production was drastically altered for CetZ1 and CetZ2 knockout strains (Supplementary Figure 18). RNA could also be isolated from EVs of this strain, and the size distribution of EV-associated RNA was nearly identical when compared to the parental strain.

Previous studies in Bacteria have shown that destabilization of the cell envelope results in a ‘hypervesiculation’ phenotype [5, 6]. To investigate whether changes in cell envelope stability would similarly affect EV production in *H. volcanii*, we assessed EV production in an *aglB* knockout strain. Cells lacking AglB are unable to N-glycosylate the S-layer glycoprotein and absence of AglB results in enhanced release of the S-layer glycoprotein [7]. Thus, deletion of this protein causes a destabilization of the structural integrity of the cell envelope. Indeed, we observed a noticeable increase in EV production from the *aglB* knockout strain during the purification process as well as by TEM (Supplementary Figure 19A-C). While we could not confirm this result when using the immunodetection-based assay for quantifying EVs (Supplementary Figure 19D and E), EV quantification by fluorescence staining revealed a 1.52-fold ($p = 0.003$) increase in EV production (Supplementary Figure 19F), indicating that CetZ1 incorporation into EVs is altered in this mutant. Interestingly, we observed a drastic change to the morphology of EVs in the mutant. The surface of EVs isolated from the AglB knockout strain was significantly different from EVs of the parental strain, appearing very fuzzy (Supplementary Figure 19B and C), likely due to the instability of the S-layer. Further, while we isolated a significantly larger amount of EVs from the mutant, the RNA yield remained the same (Supplementary Figure 19G), indicating that RNA distribution in EVs is altered.

Lipid analysis reveals differences in the relative abundance of distinct lipids between cells and EVs

To determine whether EVs selectively enclose particular lipids, we analyzed the lipid content of EVs and compared the relative abundances of different lipid compounds in EVs to that of cell membranes and total cells of *H. volcanii*. We only detected minimal differences in proportions of lipid types between EVs deriving from upper and lower bands of a density gradient (Supplementary Figure 20B), indicating that the lipid content alone is not the differentiating factor between the two subpopulations. We therefore chose to pool samples from different bands of each replicate for comparison.

Lipids with phosphate-based polar head groups were dominant across all samples. Methylated-phosphatidylglycerolphosphate-archaeol (Me-PGP-ARP) both in saturated and unsaturated form (:n) represented the most abundant lipid across samples, with relative abundances of 53.9 ± 2% in whole cells, 66.4 ± 8.81% in cell membranes and 46.8± 1.91% in EVs (Supplementary Figure 20A, Supplementary Figure 21). The ratio of unsaturated to total Me-PGP-AR abundance was identical in cells and cell membranes (28 ± 1.64% and 29 ± 3.6%), but the comparative amount of unsaturated Me-PGP-AR was lower in the vesicle fraction (11.1 ± 3%) (Supplementary Table 11). Phosphatidylglycerol-archaeol (PG-AR) was the second most abundant lipid in all fractions (18.8 ± 5.3%, 17 ± 3.7% and 32.6 ± 2.9% for cells, cell membranes and EVs respectively) and showed the highest degree of unsaturation (either 4 or 6 double bonds). The ratio of unsaturated to total PG-AR did not show a large variation between cellular (36.1 ± 4.1%), cell membrane (38.9 ± 6.4%) and extracellular fractions (31.3 ± 9%). Sulfated-diglycosyl-archaeol (S-2G-AR) showed relative abundances of 5.75 ± 4.4% (whole cells), 6.8 ± 3.9% (cell membrane) and 12.9 ± 1.5% (EVs) respectively, with negligible amounts of unsaturated lipids detected in the whole cell and cell membrane fraction.

Lipids with a neutral headgroup, such as diglycosyl-archaeol (2G-AR) or no head group, such as core-archaeol (C-AR), were detected in all fractions but showed higher relative abundances in the EV samples (2.57 ± 0.19% and 4.1 ± 1%) compared to lipid extracts from cells and cell membranes (<1.2 ± 0.2%). A notable difference was also observed for dimeric phospholipids (or cardiolipins, CL). While they contributed 20.1 ± 9.7% and 9.04 ± 8.1% of the total lipids in whole cells and cell membrane samples respectively, they were almost undetectable in the EV samples (0.91 ± 0.41%). Interestingly, we were not able to detect any extended archaeol lipids (C_{25} instead of C_{20} isoprenoidal chains) with relevant concentrations in any of the samples, despite how common they are among many haloarchaea [8].

We could not detect any lipid compounds which were only present in the vesicular fraction but not in cells or cell membranes. However, the lipid composition of EVs differed significantly to that of cells and cell membranes when comparing the relative abundance patterns of different lipid groups (Supplementary

Figure 20B, Supplementary Figure 21). The distribution between unsaturated and saturated compounds shifts towards saturated lipids from 67.5 ± 2.7% and 68.7 ± 1.7% in whole cell and cell membrane extracts, to 84.4 ± 4.7% in EVs (Supplementary Table 11). In the vesicle fraction this is likely attributable to the absence of cardiolipins and the lower abundance of unsaturated Me-PGP-ARs.

Supplementary Discussion

We were able to detect the major bilayer forming lipids PG-AR, Me-PGP-AR, S-2G-AR, C-AR, 2G-AR and cardiolipins, that were previously described for *H. volcanii* [9, 10] in all samples, albeit in different relative amounts. Me-PGP-AR and PG-AR were the two most abundant lipid species across all samples, while the cardiolipins (CL) contributed to a notable portion of the intact polar lipids (IPLs) in cells and cell membranes and were surprisingly only detected in low abundances in EVs. CLs are considered to be important for membrane curvature [11], therefore, we expected them to be essential in EVs due to the high degree of bilayer curvature in the vesicles. However, Kellermann et al [9] observed that changing extracellular Mg^{2+} levels influence CL and Me-PGP ratios in *H. volcanii* and proposed that changes to the ratio of the two compounds are used to control membrane permeability in neutrophilic haloarchaea, in response to extracellular Mg^{2+} levels. As we cultivated *H. volcanii* in medium with a constant high Mg^{2+} concentration (174 mM) it is not surprising that Me-PGP-AR was the most prominent phospholipid species across all samples. This could also explain the absence of CLs in EVs, as Me-PGP-AR may be sufficient to ensure membrane stability in the smaller-sized EVs under high Mg^{2+} concentrations. C-ARs and 2G-AR showed the opposite trend to cardiolipins, with an increase in their relative abundance in EVs compared to the cellular fraction.

Supplementary Methods

Isolation and purification of EVs

For isolation of EVs from *H. volcanii*, cultures were grown at 45 °C in minimal media with serial dilution (two times in exponential growth to OD_{600} = 0.05) before being transferred into nutrient rich media and grown at 28 °C (unless otherwise specified). EVs were isolated and purified as described in [12]. Briefly, cells were removed at late stationary (~ 144 hours growth) by centrifugation (4,500 x *g*, 40 min), and EVs were precipitated with the addition of polyethylene glycol (PEG) 6000 and incubation at 4 °C. EVs were subsequently pelleted by centrifugation (14,000 x *g*¸ 50 min, 4 °C) and after resuspending the pellet, remaining cell contaminations were removed by an additional centrifugation (14,000 x *g*, 10 min) and filtration (1 x 0.45 µm filter, 1 x 0.2 µm filter). Extracellular nucleic acids were removed with DNase I (New England Biolabs, 20 U/mL) and RNase A (New England Biolabs, 20 U/mL) [13]. The samples were further purified through an OptiPrep™ density gradient, yielding two bands containing EVs.

EV quantification

Two different quantification methods were used, because each of them proved unsuitable for some conditions tested. We assume that enclosing CetZ1 into EVs can be influenced by particular conditions. Using CetZ1 [14] as a reporter gene for detection of EVs in culture supernatants (immunodetection) was unsuitable when testing temperatures dependencies (Supplementary Figure 2A and B) and did also not reflect results that we obtained for EVs from the *aglB* knockout strain (Supplementary Figure 16D and E). The fluorescence-based method proved unsuitable for quantification of EVs in virus infected cultures, because viral particles also appeared to be stained with the fluorescent dye (Supplementary Figure 2F). P-values are calculated by unpaired, two-tailed t-test.

RNA extraction and transcriptomic analysis

RNA was extracted from cell pellets or EV pellets using TRIzol™ (Thermo Fischer Scientific). 1 mL TRIzol™ reagent was added to the pellet, homogenized by pipetting, and incubated at room temperature for 5 min. 0.2 mL chloroform was added to the sample, gently mixed via inversion, and incubated at room temperature for 10 min. The sample was then centrifuged at 4 °C for 10 min at maximal speed (~20,000 x *g*). Upper phase was transferred to a new tube, and 500 mL isopropanol was added, mixed gently by inversion, and incubated at room temperature for 10 min. The sample was then centrifuged at 4 °C for 15 min at maximal speed. The supernatant was removed and pellets washed twice with ice-cold 75% ethanol. The remaining liquid was removed and the pellet was air-dried for 10 min. Pellets were resuspended in RNase/DNase free water.

Northern blot

The Northern blotting protocol was adapted from [15]. Briefly, RNA was extracted as described above and separated on formaldehyde-MOPS agarose gels, with a final concentration of 2% formaldehyde and 2% NuSieve 3:1 agarose (Lonza). 5 µg RNA was denatured for 10 min at 70 °C with 1 X MOPS buffer (20 mM MOPS, 5 mM NaOAc, 1 mM EDTA, pH 7.0), 3.7% formaldehyde and loading dye (67 nM EDTA pH 8, bromophenol blue and xylene cyanol in deionized formamide). Samples were heat denatured for 10 min at 70 °C then placed on ice for 3 min before loading onto gel. The gel was run at 125 V for 3 to 4 hours and the RNA was then transferred to a Zeta-Probe GT membrane (Bio-Rad) by capillary action with 20 x SSC buffer (3 M NaCl, 300 mM Sodium Citrate, pH 7.0) and 2 x SSC buffer. The oligonucleotide probe is listed in Supplementary Table 2, and was labelled with [γ-^{32}P] ATP using polynucleotide kinase (Thermo Fisher).

Plasmid construction and expression of OapA

The plasmid, pTA1852 (provided by Thorsten Allers), is derived from pTA1392 [16] with a replacement of the 112 bp *Nde*I and *Not*I region containing an N-terminal 6 x His tag and a C-terminal 1 x StrepII tag with an N-terminal 7 x His tag and 2 x StrepII tag. Expression of tagged OapA ($OapA_t$) on pTA1852 is controlled by tryptophan-inducible promoter, p.tnaA.

For Expression of $OapA_t$ cultures were grown in Hv-YPC supplemented with 200 µg/mL tryptophan at 28 °C until OD_{600} of approximately 1. Cultures were then supplemented with tryptophan by adding 18% BSW containing 5 mg/mL tryptophan (final concentration of 450 µg/mL tryptophan). Cultures were grown for 2 hrs at 28 °C before EVs were quantified as described.

Affinity purification of $OapA_t$ was modified from [17]. Cells from 500 mL culture were pelleted (11,000 x *g*, 40 min) and resuspended in 7 mL Binding Buffer (20 mM HEPES pH 7.5, 2 M NaCl, 1 mM PMSF). Cells were lysed by sonication (6 x 30 seconds at 35% amplitude) on ice, and treated with 20 µL DNase I (New England Biolabs, 20 U/mL) for 1 hr at 28 °C. Lysates were centrifuged (20,000 x *g*, 15 min, 4 °C) and filtered through 0.8 µm, 0.45 µm and 0.22 µm pore-size filters. The remaining flow through was incubated overnight with 1 mL Strep-Tactin® Sepharose® beads (iba-lifesciences) equilibrated with Binding Buffer and applied to a Poly-Prep chromatography column (Bio-Rad). Flow through was run twice on the column, and the column was then washed 5 x with Binding Buffer. The column was then incubated with 3 mL Elution Buffer (Binding buffer with 5 mM D-desthiobiotin) for 30 min, and flow through was concentrated using Vivaspin® 500 centrifugal concentrator (10,000 MWCO, Sartorius). Expression of $OapA_t$ was then confirmed using Western blot (Supplementary Figure 11C).

Lipid extraction and analysis

For the total cell and cell membrane fraction, three biological replicates of cells pellets from *H. volcanii* cultures were dissolved in DBCM2 salt solution [18] and half of each replicate was used for total cell analysis and half for cell membrane extraction. For extraction of cell membranes, the dissolved cell pellet was sonicated with a microtip sonicator (MS 73 Sonoplus, Bandelin electronic, Germany) 3 times for 30 s on ice at 35% output. The lysate was treated with DNase I (30 min at 28°C, 10 µL per mL), and spun down (8,000 x *g*, 30 min, 4 °C) to remove cell debris. Cell membranes were pelleted from the supernatant by ultracentrifugation (248,000 x *g* for 15 min) and dissolved in DBCM2 salt solution. EVs from three biological replicates (200 mL cultures) were treated with DNase and RNase and purified with an Optiprep™ gradient (4 hr at 150,920 x *g*). The resulting EV bands were extracted separately from gradients. The samples were concentrated (Vivaspin 6, 100,000 MWCO PES, Sartorius, Germany) at 4 °C and washed twice with DBCM2 salt solution. Each gradient band was concentrated to 900 µL, from which 3 x 300 µL technical replicates were aliquoted.

For lipid extraction, samples in DBCM2 salt solution were sonicated for 1 h in an ice-cooled ultrasonication bath and treated with a protocol based on [19]. Phase separation after the final centrifugation resulted in an upper lipid-containing organic phase, a lower metabolite-containing aqueous phase and a protein-containing pellet. The separate phases were isolated into combusted glass LC-MS vials, dried under constant N_2 flow and stored at -20 °C until further analysis. Three 300 µL aliquots of sterile DBCM2 salt solution were treated with the same protocol as negative controls.

For ultrahigh performance liquid chromatography (UHPLC) coupled to mass spectrometry (MS) analysis, dried samples were resuspended in a solvent mixture of dichloromethane:methanol (1:9). Measurements were performed on a Dionex Ultimate 3000 RS UHPLC system coupled to a maXis ultrahigh-resolution quadrupole time of flight tandem mass spectrometer (Q-TOF MS, Bruker Daltonics). Separation of archaeal lipids was achieved on a Waters Acquity UHPLC BEH C18 column (1.7 µm, 2.1 x 150 mm) at 65 °C using reverse phase chromatography [20]. Briefly, a 26 min gradient was run at a flow rate of 400 µL min^{-1} beginning with 100% A (held for 2 min), followed by an increase to 15% B within 0.1 min and ramping to 85% B in 19 min, followed by 8 min re-equilibration with eluent B. Eluent A was MeOH:H_2O (85:15) and eluent B was IPA:MeOH (50:50), both with addition of 0.04% HCO_2H and 0.1% NH_3. Analysis was performed in positive ionization mode, scanning from m/z 100 to 2000. MS2 scans were obtained in data dependent mode.

Output data were analyzed with the manufacturer's software (DataAnalysis 4.4.2, Bruker Daltonics). Lipid compounds were identified based on retention time, fractionation pattern and exact masses [21, 9, 22]. Several technical replicates were measured for each sample type, of which representative replicates were selected for each biological replicate. Since the EV samples showed minimal differences in lipid distribution between bands after ultracentrifugation (Supplementary Figure 20B), gradient bands were pooled for each biological replicate for further analysis. The samples were compared with respect to their relative abundance distributions without absolute quantification.

The relative abundances were normalized per replicate and averages for each fraction were calculated from three biological replicates (total cells and cell membrane fraction) and from the upper bands after density gradient centrifugation from three biological replicates (EV fraction). Figures were created in R Statistical Software (v4.1.2; R Core Team 2021) with the ggplot2 [23], plyr [24] and dplyr packages [25].

Identification of arvB and arvC homologs

For analysis of the distribution of *arvB* and *arvC* we examined the proteins encoded by the first and second gene downstream of the 1,666 *arvA* genes. We retrieved all proteins that were annotated as COG3365 and COG3364 for the gene at the first and second position downstream of *arvA*, respectively. COG3365

(Uncharacterized conserved protein, DUF2073 family; https://www.ncbi.nlm.nih.gov/research/cog/cog/COG3365/) and COG3364 (Predicted nucleic acid-binding protein, contains Zn-ribbon domain; https://www.ncbi.nlm.nih.gov/research/cog/cog/COG3364/) are both predominantly found in Archaea. This selection resulted in a set of 1,374 proteins for the first gene downstream COG3365, and 948 proteins for the second gene downstream (COG3365). To assess whether there were other homologs of these proteins in archaeal and bacterial genomes, and to determine whether we had missed homologs downstream of *arvA* by relying on COG annotation, we then used these protein sets as reference databases in a DIAMOND search as described for ArvA. For ArvB (first gene downstream, COG3365) this search resulted in 1,749 hits that were inspected using an alignment score ratio approach as described for ArvA. All hits met the selection criteria, and were used for further analysis. For ArvC (second gene downstream, COG3364) this search resulted in 14,474 hits. After filtering of the hits using an alignment score ratio approach as described for ArvA, 1,650 sequences were selected for further analysis. For both *arvB* and *arvC* the presence or absence in genomes containing *arvA* was then determined, as well as the genomic location relative to *arvA*.

Supplementary Figures

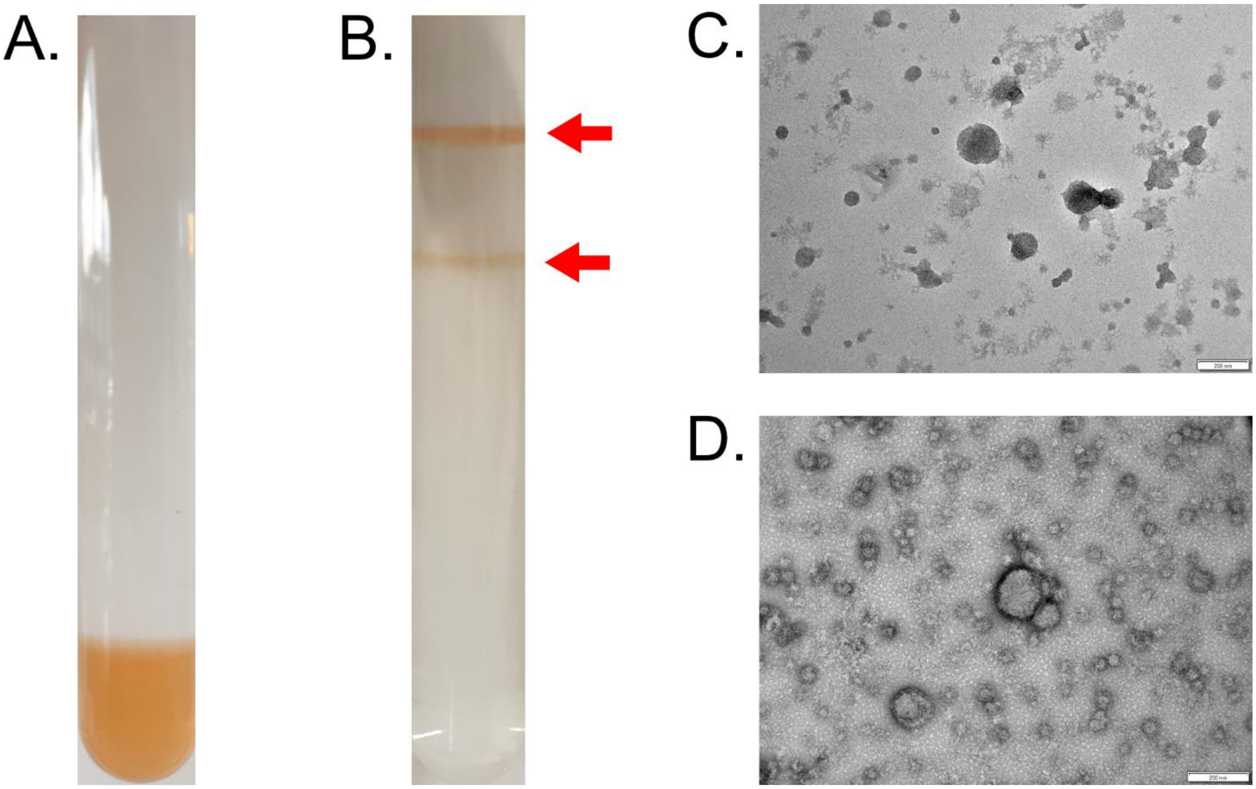

Supplementary Figure 1. Purification of *H. volcanii* H26 EVs by Optiprep™ density gradient purification. Gradient before **(A)** and after **(B)** ultracentrifugation. Red arrows indicate upper and lower band. Transmission electron micrograph of EVs isolated from upper **(C)** and lower **(D)** bands. Size bar: 200 nm.

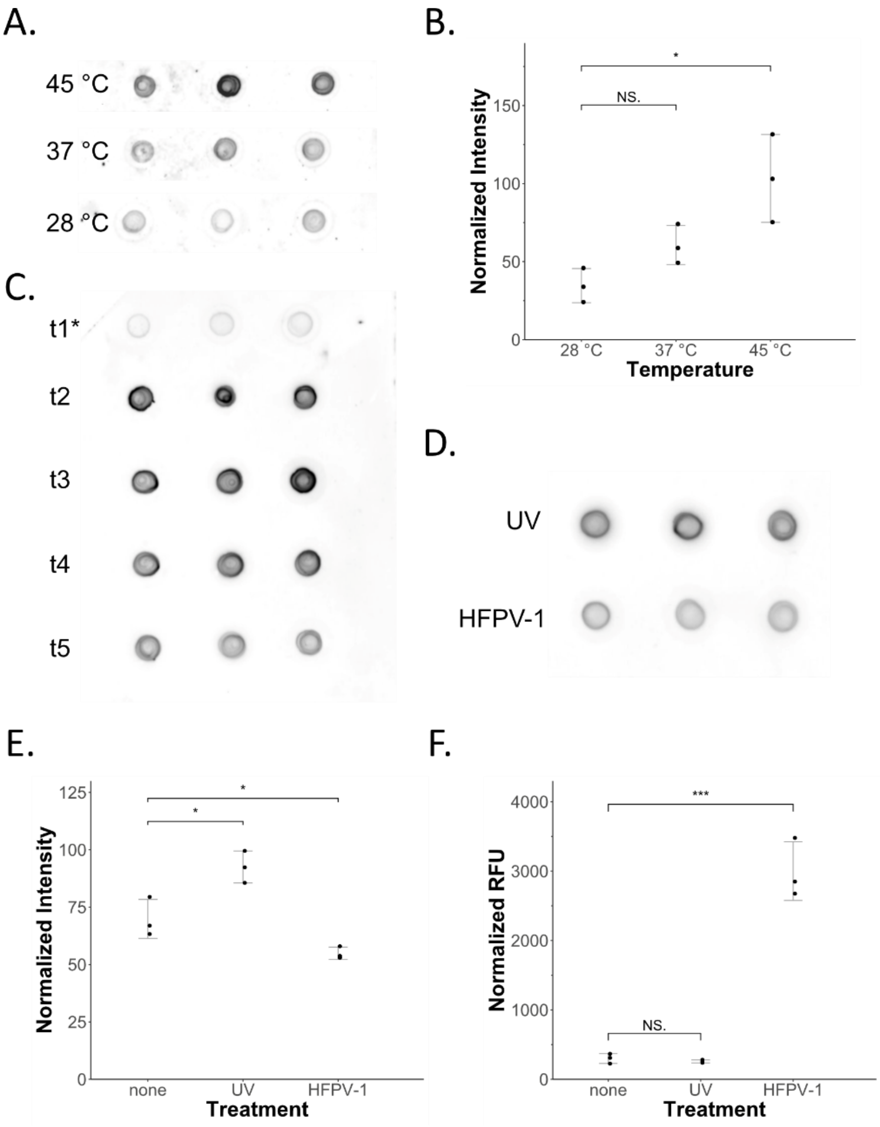

Supplementary Figure 2. EV quantification of *H. volcanii* cultures grown under different conditions and through growth. (A) Spot blot for quantification of EVs in *H. volcanii* culture supernatants grown at 45, 37 and 28 °C. **(B)** Plot representing EV production in different temperatures quantified by immunodetection (original spot blot in **A**). Intensity of spot blot signal was normalized to OD_{600} of culture. **(C)** Spot blot for quantification of *H. volcanii* grown at 28 °C with time points taken at 45.3, 68.5, 94, 118.3, and 140.5 hours. Asterisk on time point 1 represents sample diluted by a factor of 2. **(D)** Spot blot for quantification of *H. volcanii* grown with either UV or viral stress. **(E)** Plot representing EV production with either UV or viral stress, quantified by immunodetection (original spot blot in **D**). **(F)** Plot representing EV production with either UV or viral stress, quantified by fluorescence labeling. Significance is indicated above the graph (NS. indicates "not significant", * indicates "$p \leq 0.05$", *** indicates "$p \leq 0.001$"). Spot blot images were modified by subtracting background on ImageJ (rolling ball radius = 25).

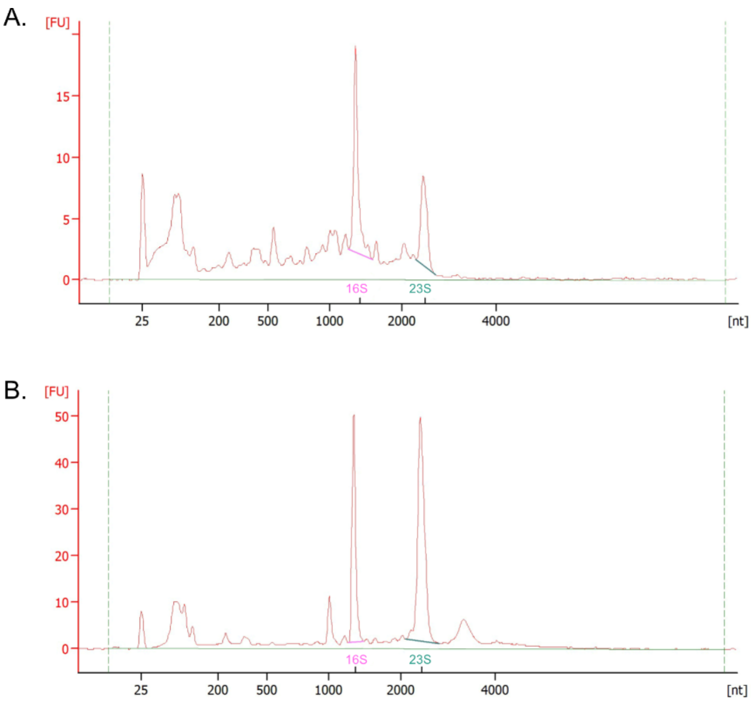

Supplementary Figure 3. Electropherograms of EV-associated RNA (A) and cellular RNA (B). Capillary electrophoresis demonstrates differences in size distribution between RNA isolated from EVs and cells of *H. volcanii*.

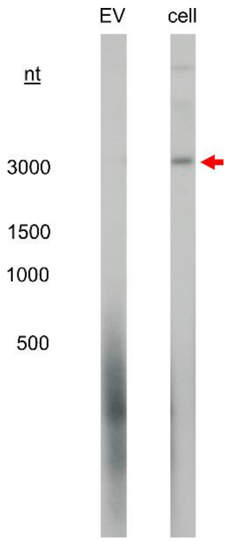

Supplementary Figure 4. Northern blot with EV and cellular RNA probed for HVO_2072. Red arrow indicates full-length transcript. Northern was conducted in duplicates, but only one replicate is presented here.

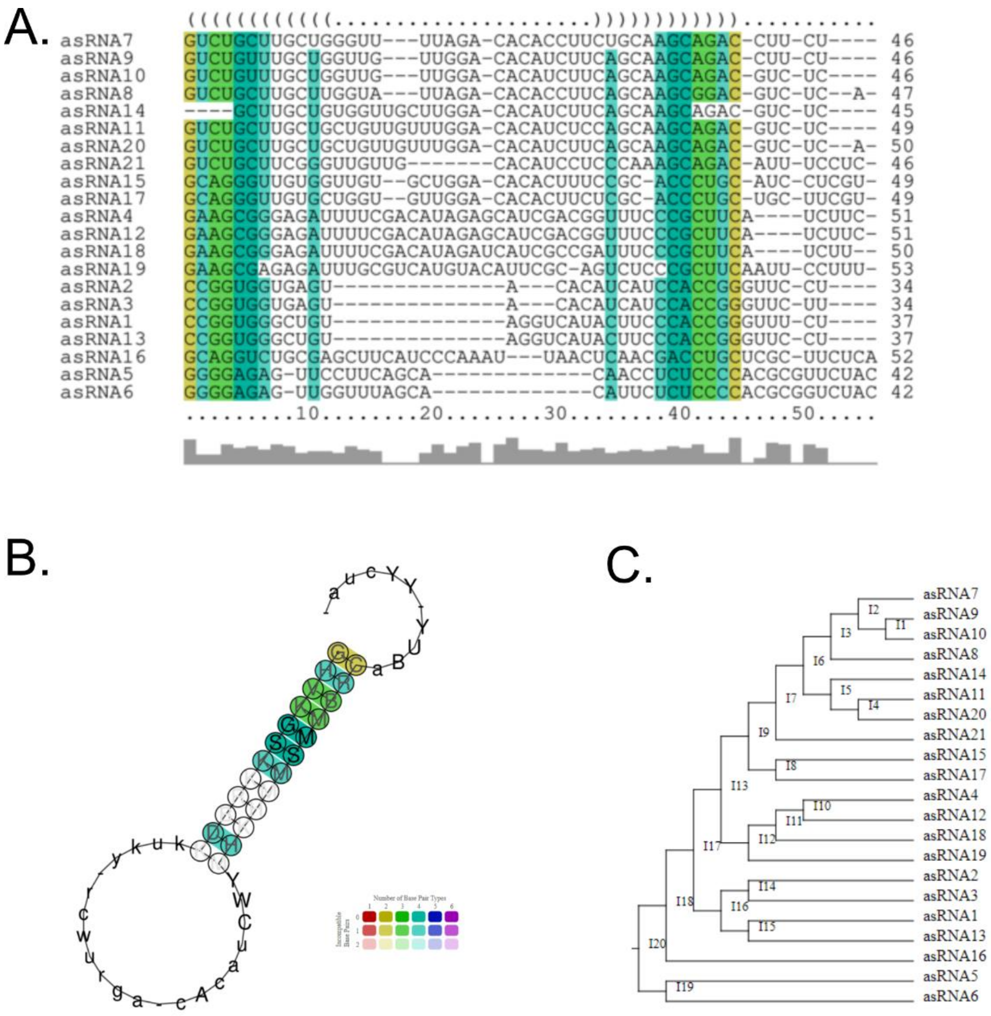

Supplementary Figure 5. Secondary structure alignment prediction of EV-associated asRNA from LocARNA [26–28]. (A) Sequence alignment with RNAalifold consensus structure. **(B)** Predicted consensus secondary structure with legend. **(C)** Hierarchical clustering based on similarities of sequences.

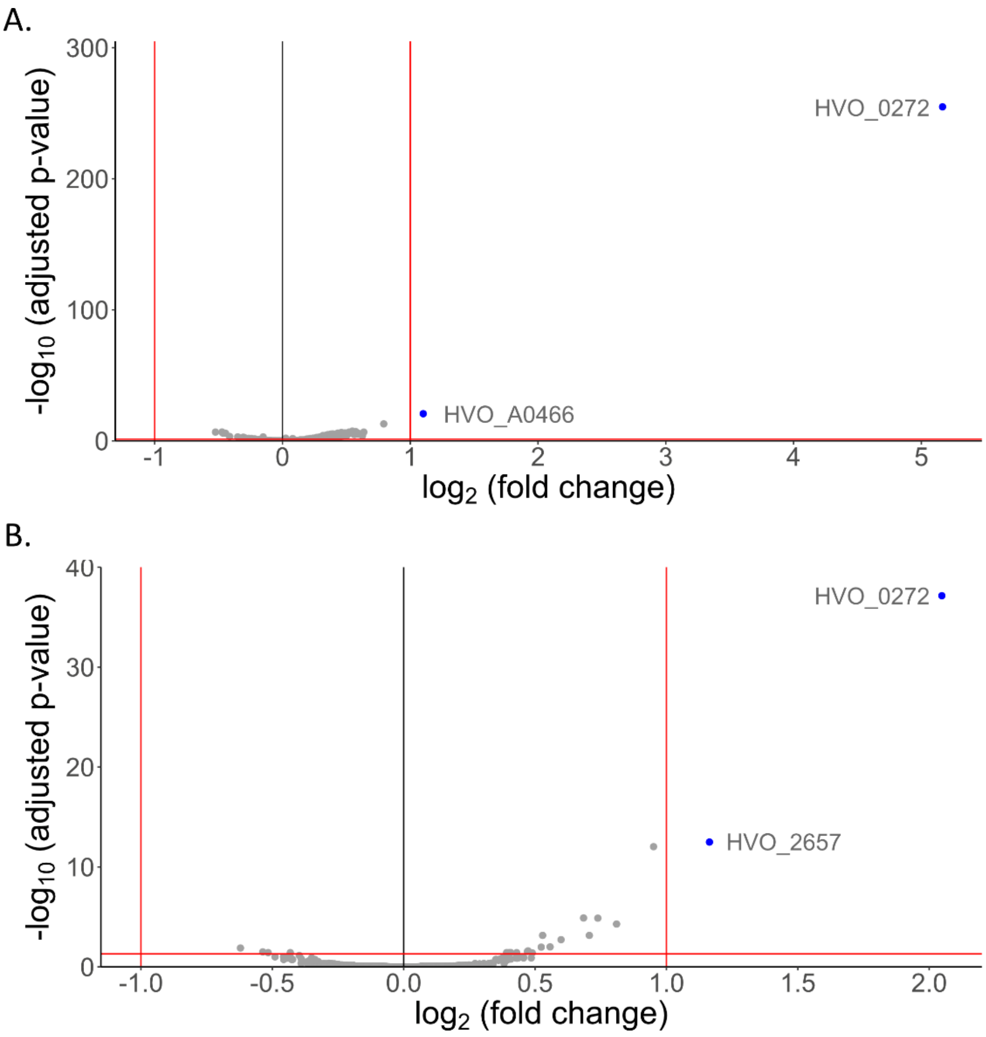

Supplementary Figure 6. Volcano plots of intracellular and EV-associated transcripts comparing cultures infected with HFPV-1 and uninfected control cultures. (A) Differential expression of transcripts in EVs from infected versus uninfected cultures. **(B)** Differential expression of intracellular transcripts from infected versus uninfected cultures. Cells and EVs were isolated at late stationary phase of growth. Volcano plots only depict transcripts that had an average TPM greater than 10 in either infected or uninfected samples. Red asymptotes indicate thresholds for enrichment ($p = 0.05$ and $|\text{fold change}| = 2$).

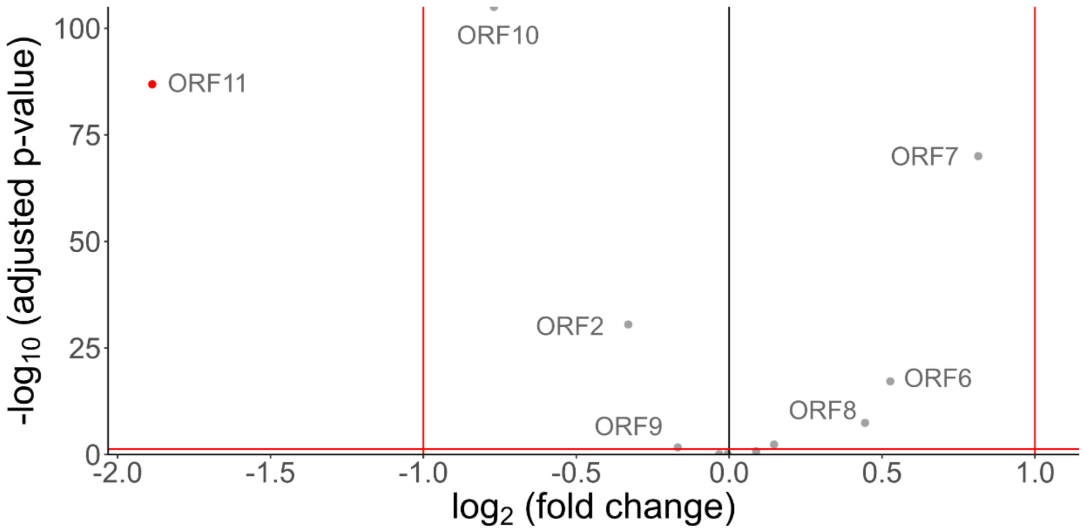

Supplementary Figure 7. Volcano plot comparing viral transcript abundance between EV-associated RNA and cellular RNA. RNA isolated from EVs and cells of cultures infected with HFPV-1 during late stationary phase. Red asymptotes indicate thresholds for enrichment ($p = 0.05$ and $|\text{fold change}| = 2$).

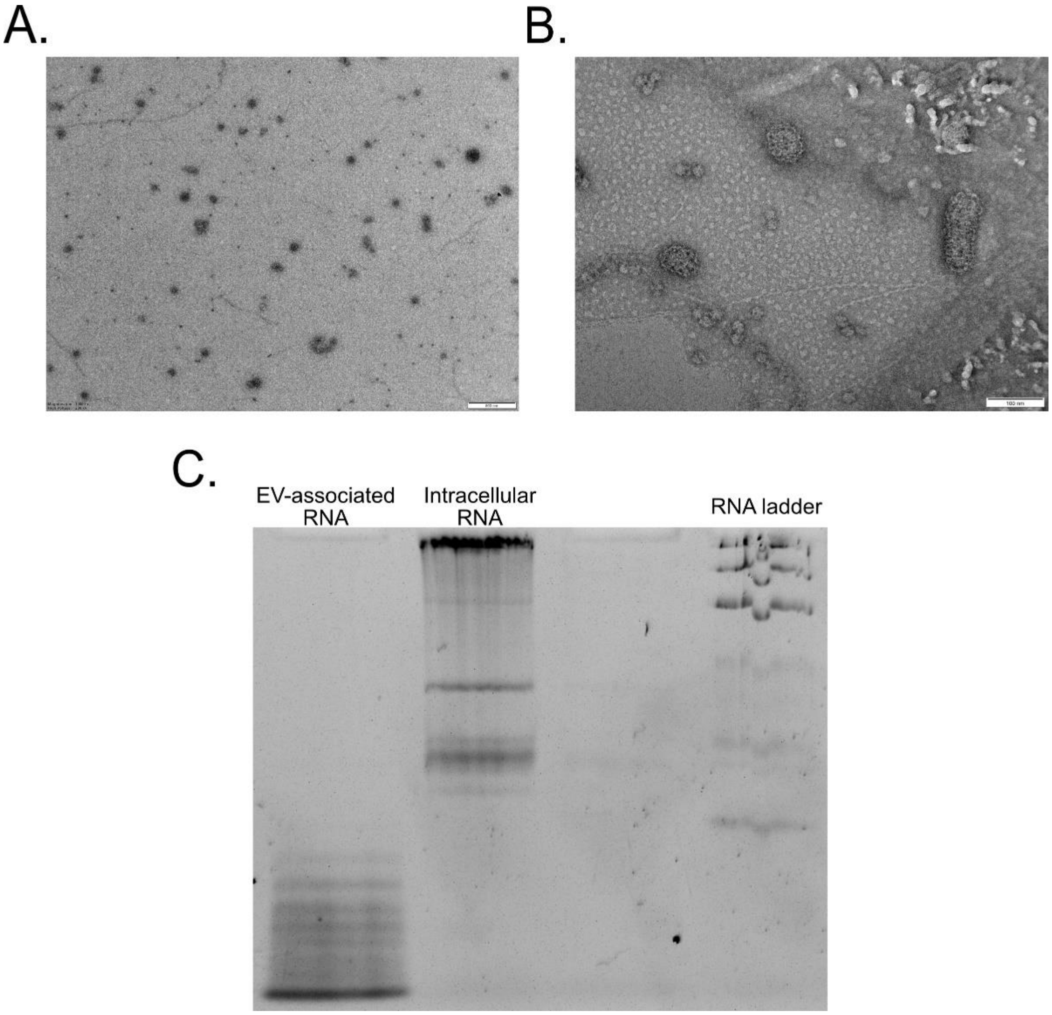

Supplementary Figure 8. EVs from other haloarchaea. (A) Transmission electron micrograph of purified EV from *Hbt. salinarum.* Scale bar: 500 nm. **(B)** Transmission electron micrograph of purified EVs from *Hrr. lacusprofundi*. Scale bar = 100 nm. **(C)** RNA extracted from gradient purified EVs and cells of *Hrr. lacusprofundi* on a 12.5% Urea-PAGE gel.

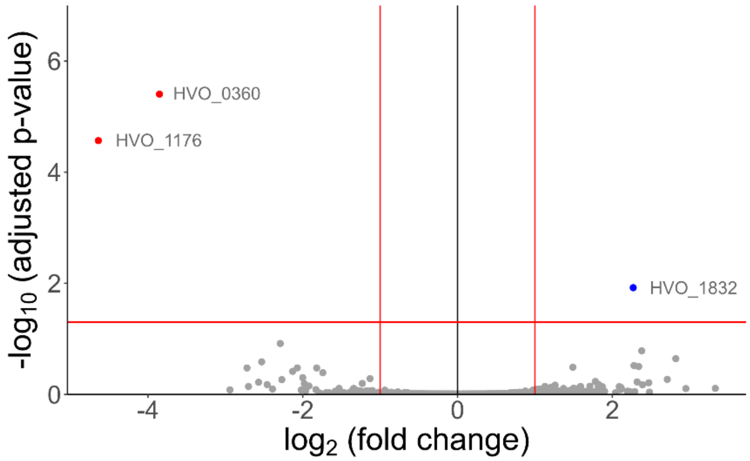

Supplementary Figure 9. Volcano plot comparing protein content from *H. volcanii* EVs isolated from upper and lower bands of Optiprep™ density gradient. EVs were isolated from three replicates at stationary phase of growth. Red asymptotes indicate thresholds for enrichment (p = 0.05 and |fold change| = 2). Differential protein abundancies and adjusted p-values were calculated by DEP (see methods).

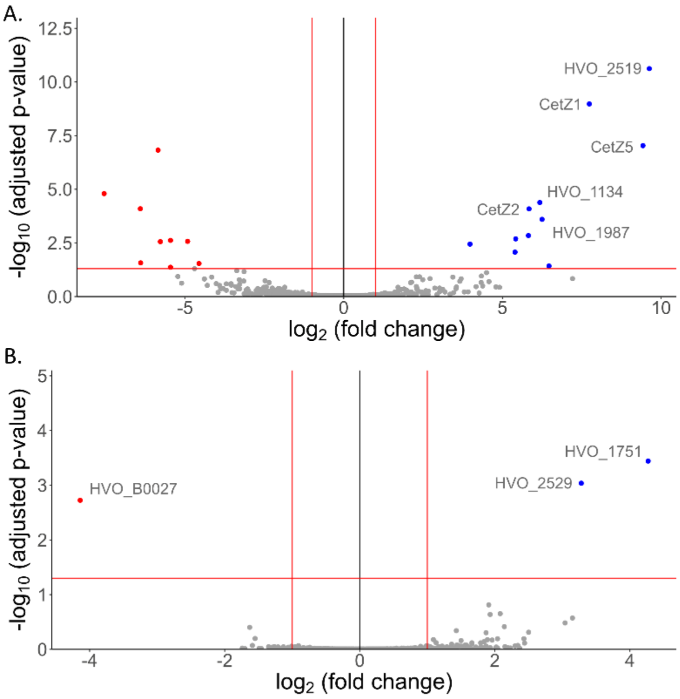

Supplementary Figure 10. Volcano plots depicting differences in protein abundance from UV-treated cultures. (A) Proteins isolated from EVs of UV-treated cultures are compared to their respective cell membrane protein content. **(B)** EV-associated proteins from UV-treated cultures are compared to EV-associated proteins from untreated cultures. Raw data found in Supplementary Table 9. Red asymptotes indicate thresholds for enrichment (p = 0.05 and |fold change| = 2).

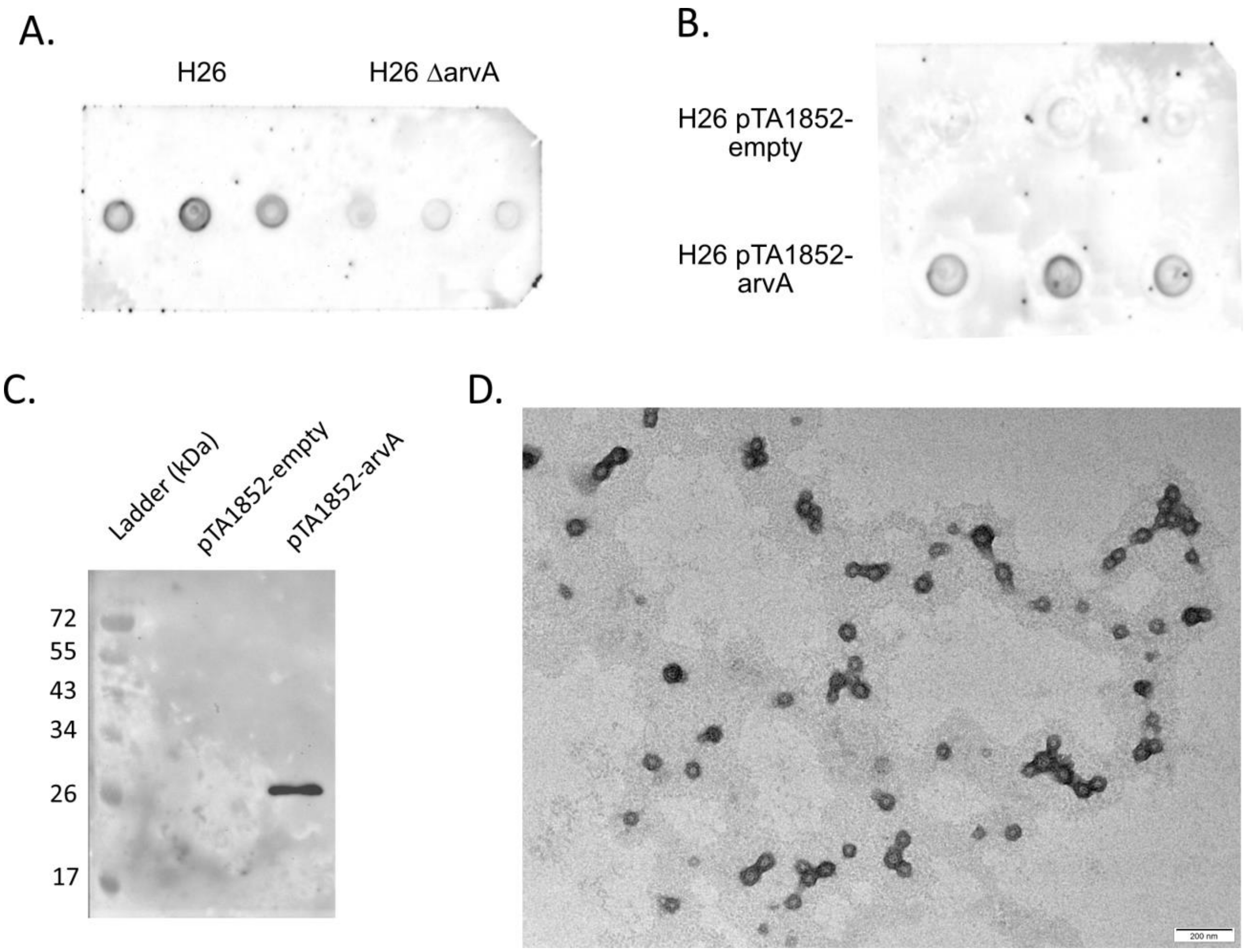

Supplementary Figure 11. Knockout and overexpression of ArvA. Original spot blots of EV production from culture supernatants of strains with knockout **(A)** and overexpression **(B)** of ArvA (quantifications found in Figure 4A and B). Spot blots were modified by subtracting background on ImageJ (rolling ball radius = 25). **(C)** Western blot with an anti-Strep tag antibody on affinity purified ArvA expressed in H26, compared to an affinity purification from H26 with the empty vector (see methods). **(D)** Transmission electron micrographs of EVs isolated from strains overexpressing ArvA. Scale bar 200 nm.

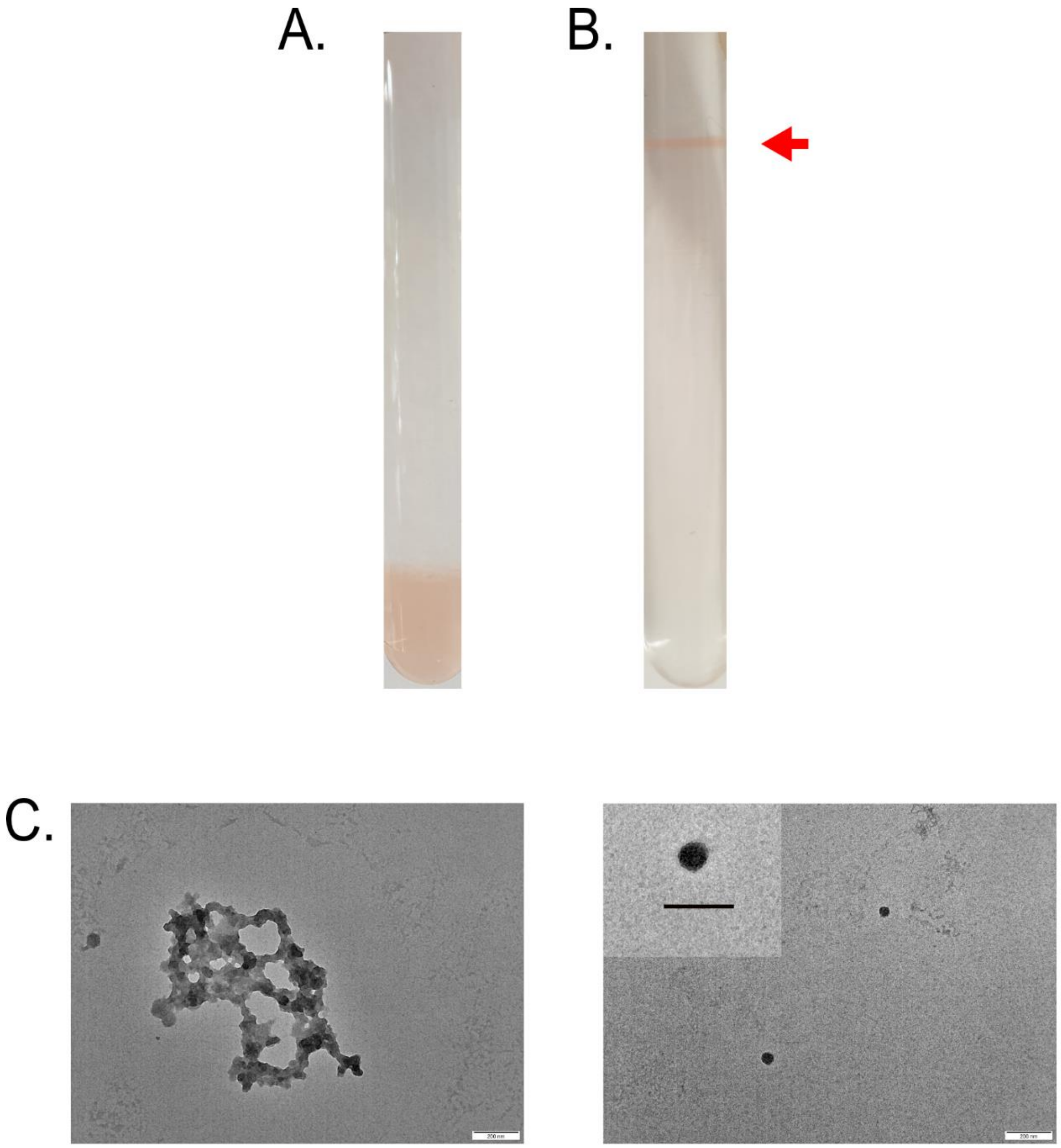

Supplementary Figure 12. EV preparations from ArvA knockout strain. EVs **(A)** before and **(B)** after ultracentrifugation. Red arrow indicates where particles concentrated. For comparison with parental strain, see Supplementary Figure 1A. **(C)** Transmission electron micrographs of supernatant from ArvA knockout strain cultures. Samples were negatively stained with 2% uranyl acetate. Scale bar 200 nm.

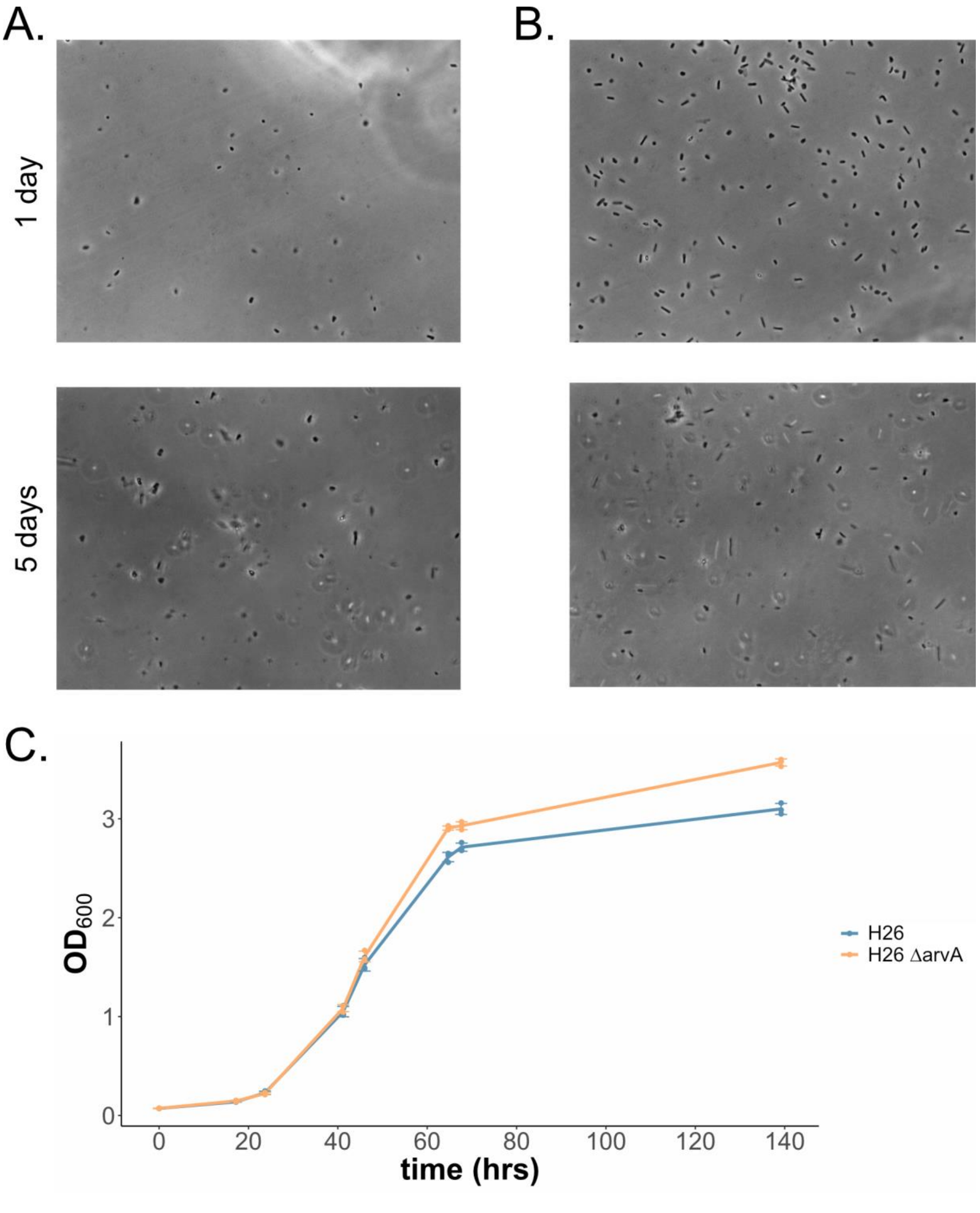

Supplementary Figure 13. Phenotypes of ArvA knockout strain in comparison to parental strain. Phase contrast microscopy images of cells from OapA knockout strain **(A)** and parental strain **(B)** after 1 day (top) and 5 days (bottom) of growth. Samples were fixed with 1% glutaraldehyde and visualized with Axiophot Zeiss microscope. **(C)** Growth curve of parental strain (H26) and OapA knockout strain (H26 ΔoapA). Error bars represent standard deviation from three replicates.

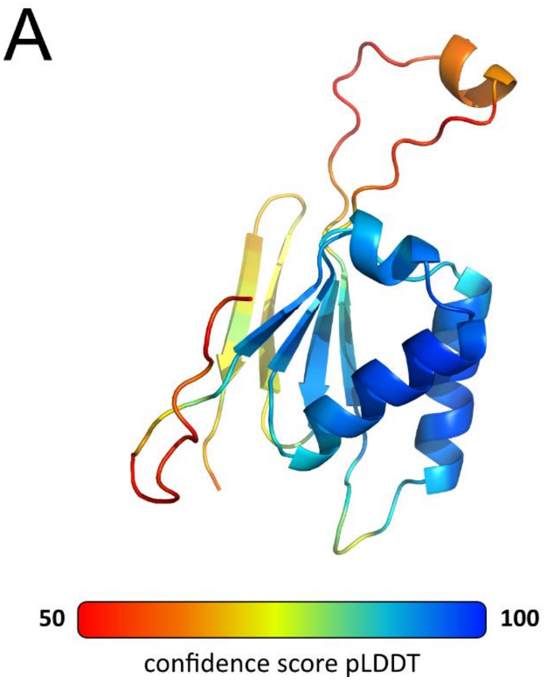

Supplementary Figure 14. Structural prediction of ArvB (A) and alignment with SepF (B). Structure was predicted using AlphaFold v2 [29], and color-coded depending on confidence measured by predicted local-distance difference test (pLDDT). The predicted structure was used to identify structurally similar proteins using DALI [30], which identified SepF family proteins (IPR038594) to be structurally similar. ArvB was aligned to SepF (3ZIG**,** shown in green) with a root mean square deviation (RMSD) value of 2.962 (210 to 210 atoms).

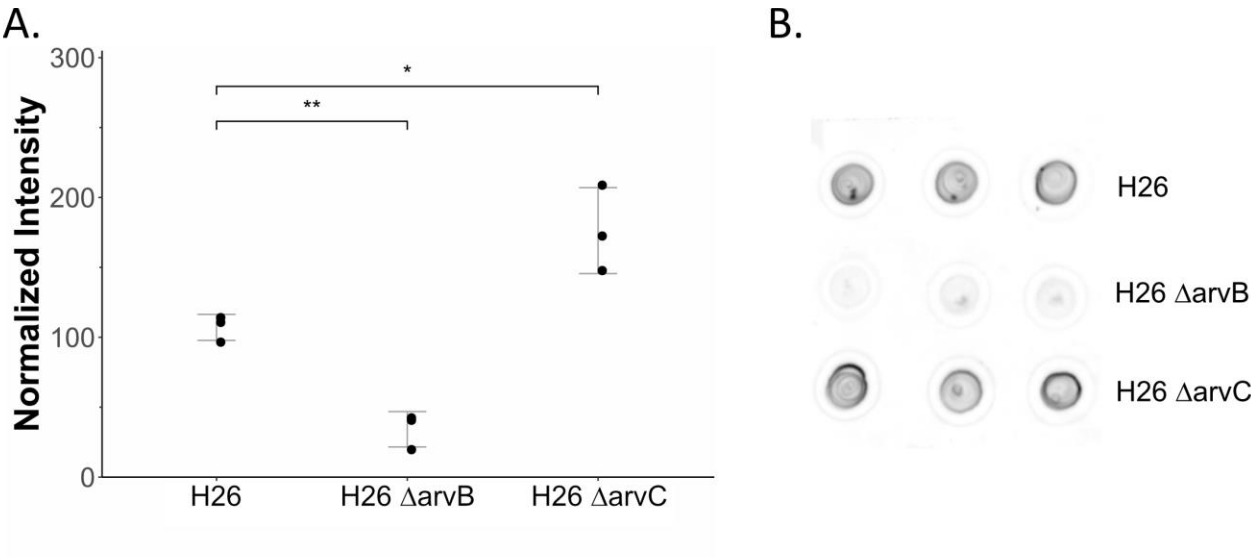

Supplementary Figure 15. EV production in knockout strains of downstream genes of *arvA*. (A) Immunodetection-based quantification of EV production from the supernatant of knockout strains of ArvB and ArvC. **(B)** Original spot blot for **(A)**. Spot blots were modified by subtracting background on ImageJ (rolling ball radius = 25).

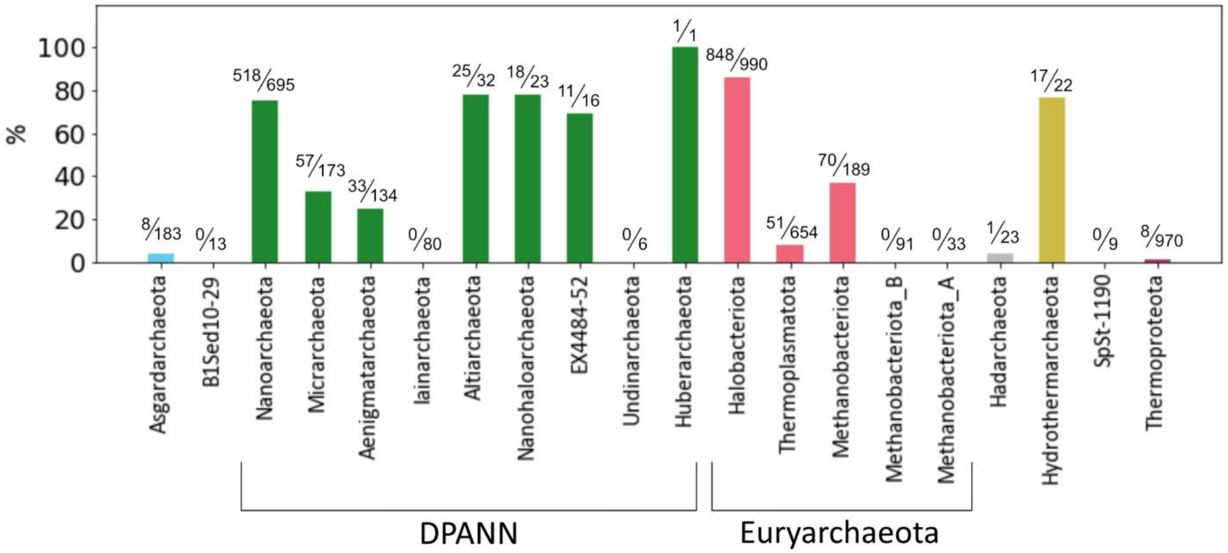

Supplementary Figure 16. Archaeal vesiculating GTPase, ArvA, is conserved among specific archaeal lineages. Percentage of species identified containing an ArvA homolog within each phylum. Archaeal phyla denoted according to Genome Taxonomy Database [31]. B1Sed10-29, EX4884-52 and SpSt-1190 have been suggested to be named *Candidatus* Oferarchota, *Candidatus* Axalarchota and *Candidatus* Iduparchota, respectively [32]. Fraction above each bar denotes the number of species identified over the number of species surveyed.

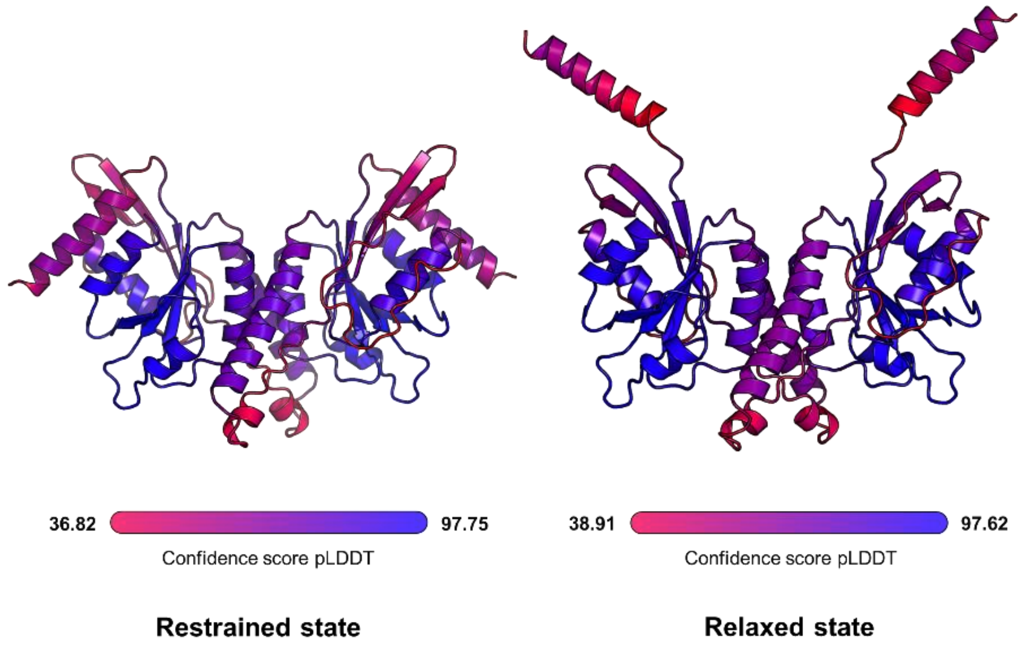

Supplementary Figure 17. Predicted structures of the ArvA homodimer. Structures were predicted using AlphaFold2 [29, 33], which predicted two distinct conformations of the N-terminal α-helix. Color-coded denotes confidence measured by predicted local-distance difference test (pLDDT).

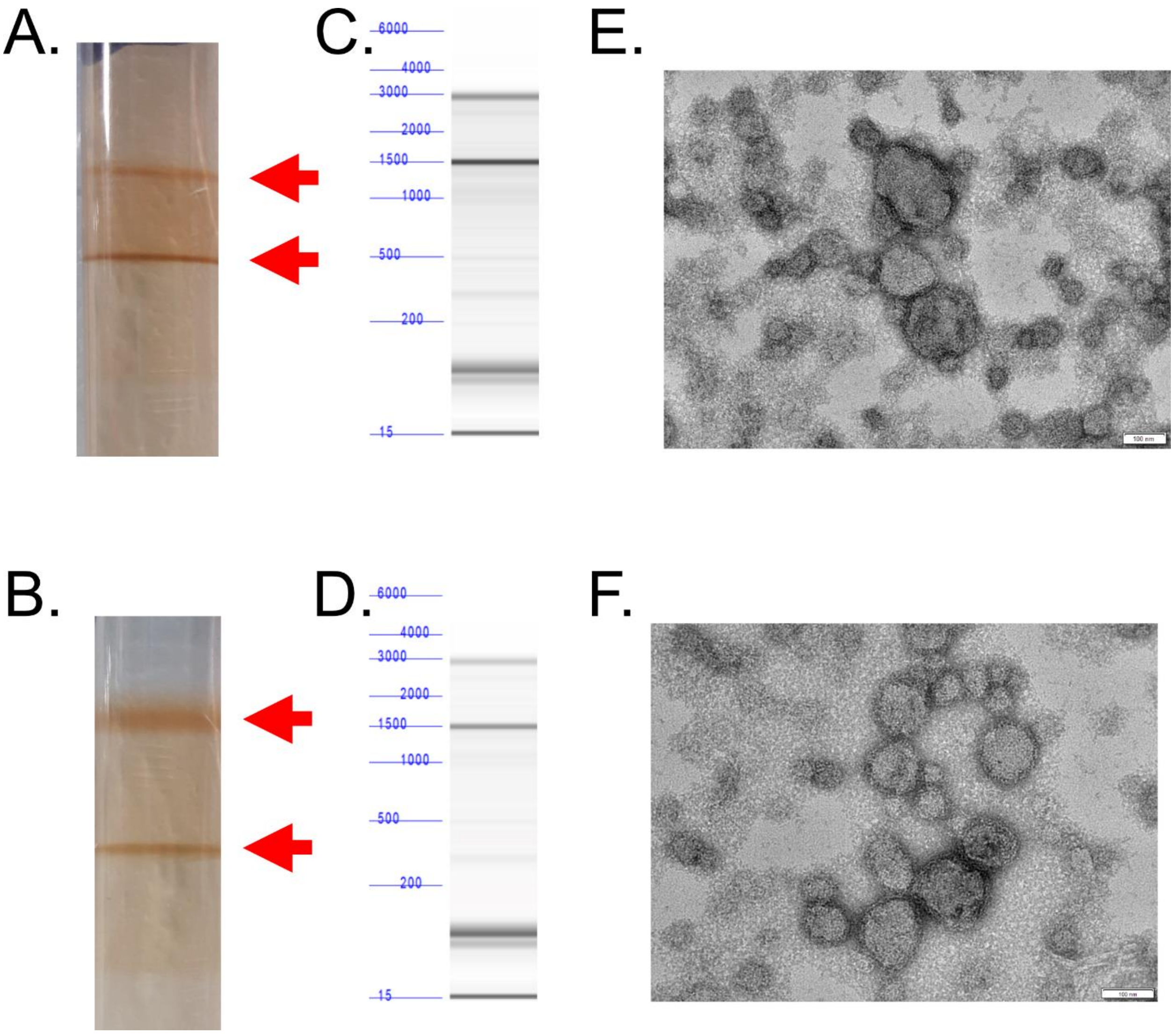

Supplementary Figure 18. EVs isolated from CetZ1 and CetZ2 knockout strains. Optiprep™ density gradients of EVs isolated from CetZ1 **(A)** and CetZ2 **(B)** knock out strains after ultracentrifugation. Red arrows indicate the upper and lower bands where particles had concentrated. **(C and D)** RNA was isolated from both strains and run on a fragment analyzer to observe the size distribution. For comparison to wild type, see Figure 2. **(E and F)** EVs could also be observed through TEM. Scale bars: 100 nm.

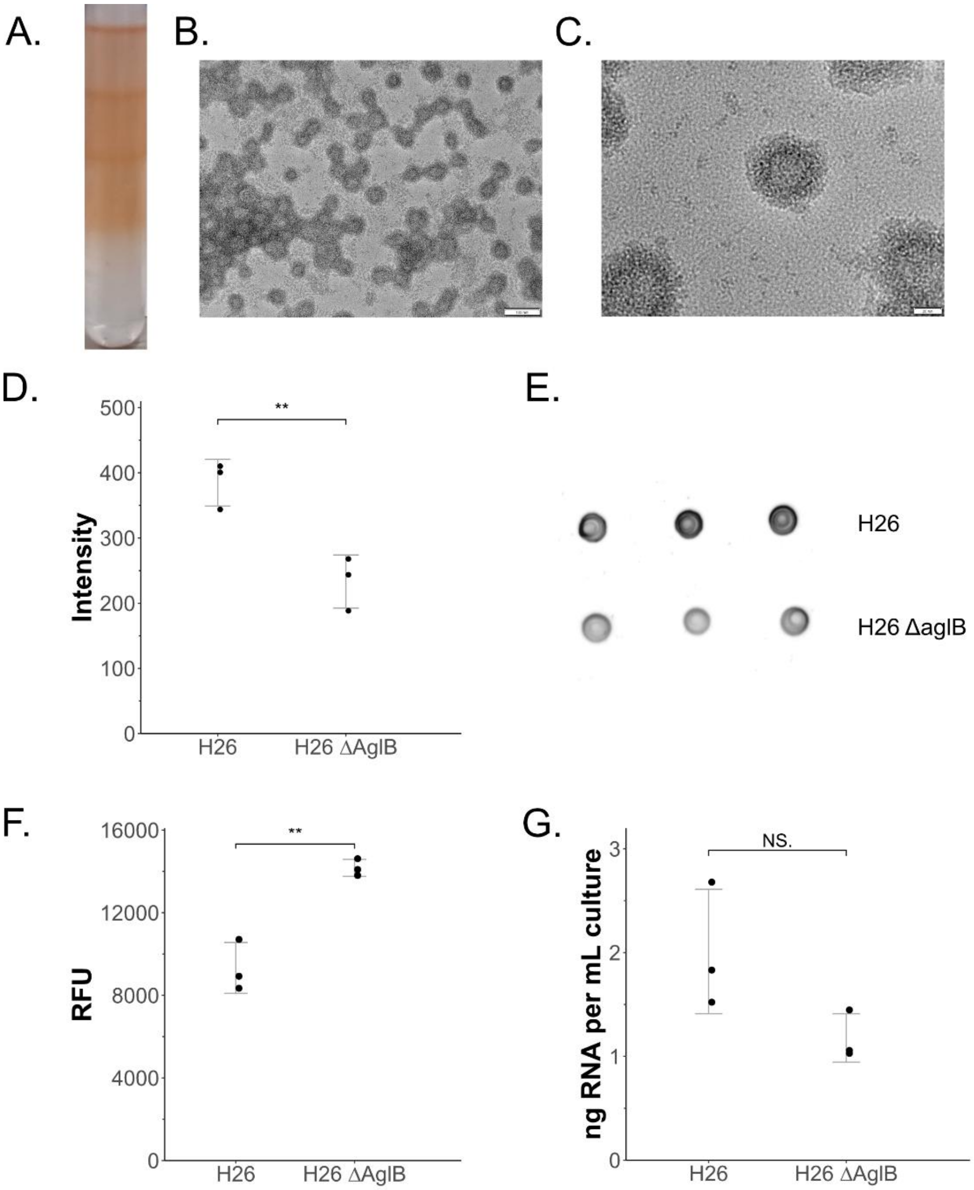

Supplementary Figure 19. Phenotypes of EVs from AglB knockout strain. (A) Bands of concentrated EVs after ultracentrifugation in Optiprep™ density gradient. Transmission electron microscopy of EVs isolated from AglB knockout strain with size bar 100 nm **(B)** and 20 nm **(C)**. **(D)** Quantification of EV production of AglB knockout strain compared to parental strain by immunodetection and the corresponding spot blot **(E). (F)** Quantification of EV production from cultures of AglB knockout strain compared to parental strain by fluorescence staining, measured in relative fluorescence units (RFU) **(G)** Quantification of EV-associated RNA in culture supernatants of AglB knockout strain compared to parental strain normalized to OD (nm = 600). Error bars represent one standard deviation from the mean value. Significance calculated using a two-tailed t-test (** indicates "$p < 0.01$, NS indicates "not significant").

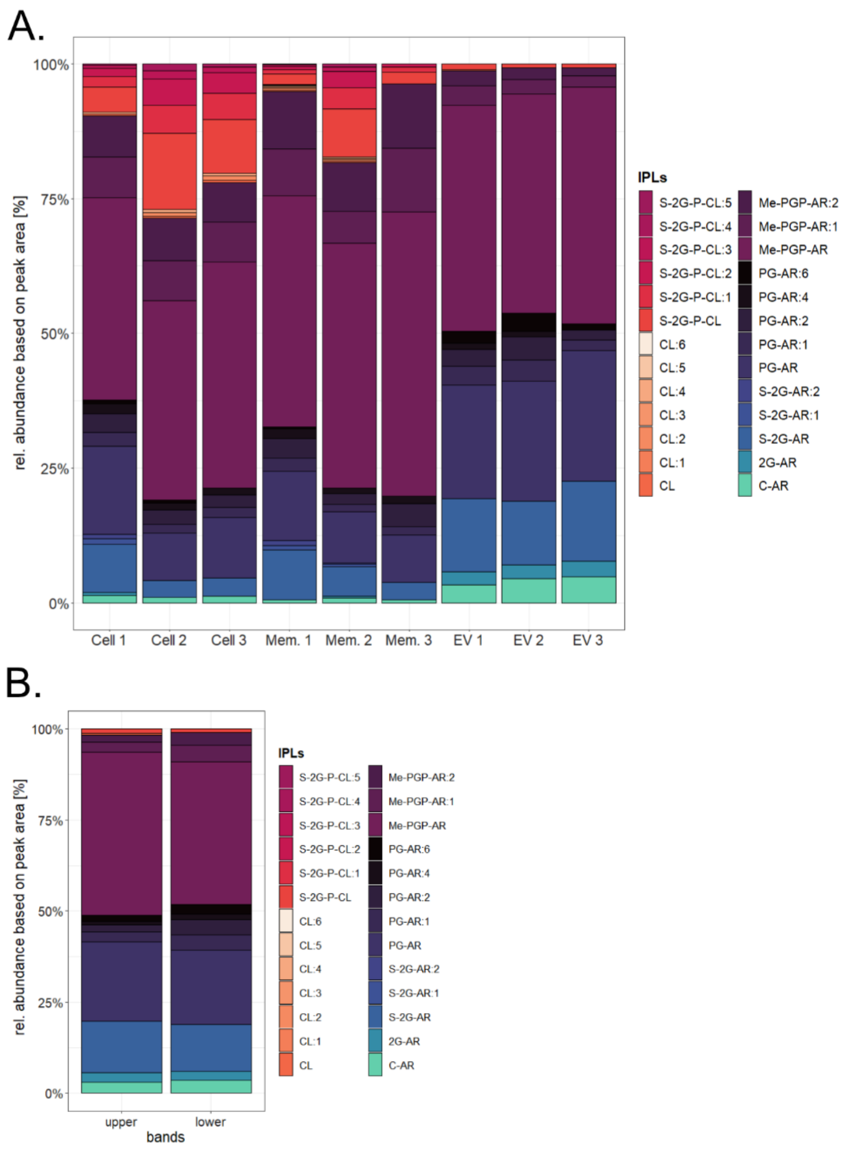

Supplementary Figure 20. Distribution of lipid compounds comparing whole cells, cell membranes and EVs of H. volcanii. (A) Individual replicates used to calculate the average relative abundances in Figure 6. Cell 1-3: whole cells, Mem. 1-3: membrane fraction and EV 1-3: extracellular vesicles after ultracentrifugation in Optiprep™ density gradients and bands pooled together for each biological replicate. **(B)** The lipid distribution in the upper (left column) and lower band (right column) after ultracentrifugation from one biological replicate. Relative abundances were calculated based on the peak area of the most abundant adduct for each compound. Lipids were identified based on their retention time, fractionation pattern and exact mass.

Compound abbreviations: AR = archaeol (C20-C20 isoprenoidal chains), CL = cardiolipin, :nUS = lipid with n number of unsaturations, UK = unknown compound. Lipids with neutral headgroups: 1G = monoglycosyl, 2G = diglycosyl, C-AR = core-AR. Lipids with anionic headgroups: PGP-Me = phosphatidylglycerophosphate methyl esters, PG = phosphatidylglycerol, S-2G =sulfated diglycosyl, S-GP = sulfoglycophospho, 2-PGLY = diphosphoglycerol.

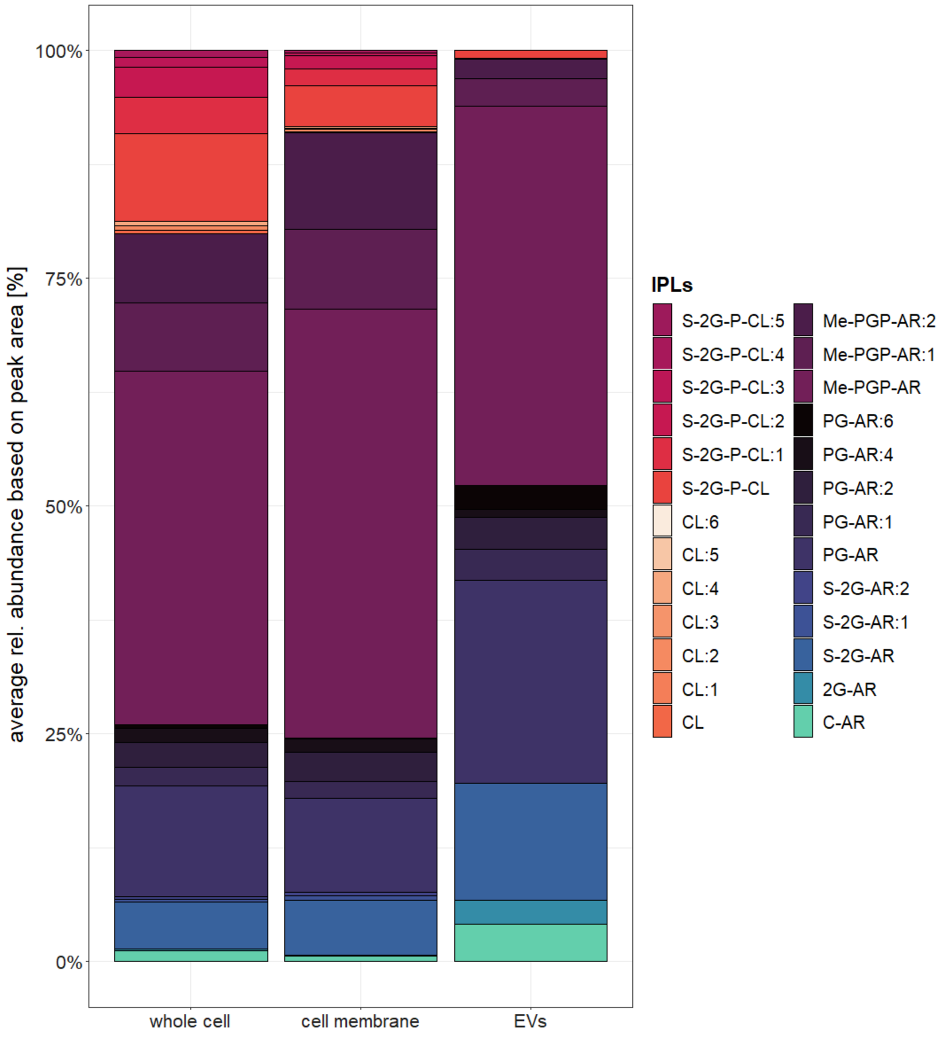

Supplementary Figure 21. Average distribution of lipid compounds comparing whole cells, cell membranes and EVs of *H. volcanii*. The average (n = 3) relative abundance of lipids was calculated for each preparation; whole cells, cell membrane and EVs based on the peak area of the most abundant adduct for each compound. The distribution in the individual samples is shown in Supplementary Figure 20A. For the EV fraction, bands after density gradient purification were pooled together from 3 biological replicates. Lipids were identified based on their retention time, fractionation pattern and exact mass.

Compound abbreviations: AR = archaeol (C_{20}-C_{20} isoprenoidal chains), CL = cardiolipin, :nUS = lipid with n number of unsaturations, UK = unknown compound. Lipids with neutral headgroups: 1G = monoglycosyl, 2G = diglycosyl, C-AR = core-AR. Lipids with anionic headgroups: Me-PGP = phosphatidylglycerophosphate methyl esters, PG = phosphatidylglycerol, S-2G =sulfated diglycosyl, S-GP = sulfoglycophospho, 2P-GLY = diphosphoglycerol.

Supplementary Tables

Supplementary Table 1: Strains used in this study

Name	Reference	Organism	Description	Media	Supplement
H. volcanii DS2	[34]	*H. volcanii DS2*	Wild type strain	HV-cab/YPC	none
H26	[35]	*H. volcanii*	ΔpyrE2	HV-cab/YPC	uracil
H26 ΔoapA	[36]	*H. volcanii*	ΔpyrE2, ΔoapA	HV-cab/YPC	uracil
H26 ΔoapB	[36]	*H. volcanii*	ΔpyrE2, ΔoapB	HV-cab/YPC	uracil
H26 ΔoapC	[36]	*H. volcanii*	ΔpyrE2, ΔoapC	HV-cab/YPC	uracil
H26 ΔaglB	[7]	*H. volcanii*	ΔpyrE2, ΔaglB	HV-cab/YPC	uracil
H53	[35]	*H. volcanii*	ΔpyrE2, ΔtrpA	HV-cab/YPC	uracil, tryptophan
H53 ΔcetZ1	[14]	*H. volcanii*	ΔpyrE2, ΔtrpA, ΔcetZ1	HV-cab/YPC	uracil, tryptophan
H53 ΔcetZ2	[14]	*H. volcanii*	ΔpyrE2, ΔtrpA, ΔcetZ2	HV-cab/YPC	uracil, tryptophan
Halobacterium salinarum	[37]	*Halobacterium salinarum*	Wild type strain	HS-Media	none
Halorubrum lacusprofundi DL18	[38]	*Halorubrum lacusprofundi DL18*	Wild type strain	DBCM2	none

Supplementary Table 2: Primer Sequences

Name	Oligonucleotide sequence 5'-3'	Description
HFPV1F	CACGAACGAGAACACCGACC	Forward primer to test infection of HFPV-1
HFPV1R	TGATGACGAATCCAACGAGCAG	Reverse primer to test infection of HFPV-1
AglB_US_F	CCGGCCAAGCTTGGTTTGCGAGCGACCCAGTCG	Forward primer for upstream flank of aglB with HindIII restriction sites
AglB_US_R	GAATTCGCCGCCCGAAGATCTTGTGACCAACAACCGCCAAG	Reverse primer for upstream flank of aglB with EcoRI and BglII restriction sites
AglB_DS_F	AGATCTTCGGGCGGCGAATTCCACGAGCCGAGACGGCGACGA	Forward primer of downstream flank of aglB with BglII and EcoRI restriction sites
AglB_DS_R	CCGGCCGGATCCGCGCGTCGCCGTGCTCGGAC	Reverse primer for downstream flank of AglB with BamHI restriction sites
csg probe	GCTGTCAGCGTCGAGGTTTCC	Northern blot probe for the 5' end of S-layer mRNA

Supplementary Table 3: Differential expression calculated for transcripts from total vs small RNA libraries of EV associated RNA. One replicate of RNA associated with EVs isolated from the upper band of a density gradient was sequenced using a total RNA library and a small RNA library. Read mapping

(90% minimum overlap identity, TPM) and differential expression (log2 ratio) performed with Geneious™ (2021.0.1). (Excel file)

Supplementary Table 4: Differential expression calculated for transcripts from EVs of upper versus lower band of a density gradient. One replicate of RNA associated with EVs isolated from the upper band and the lower band of a density gradient. Read mapping (90% minimum overlap identity, TPM) and differential expression (log2 ratio) performed with Geneious™ (2021.0.1). (Excel file)

Supplementary Table 5: Differential expression calculated for transcripts from EVs from untreated and UV treated cells. One replicate of RNA associated with EVs isolated from untreated and UV treated cells. Read mapping (99% minimum overlap identity, TPM) and differential expression (log2 ratio) performed with Geneious™ (2021.0.1). (Excel file)

Supplementary Table 6: Differential expression calculated for EV associated transcript normalized with intracellular levels. RNA was extracted from purified EVs and the respective cells in triplicates. Read mapping (99% minimum overlap identity, TPM) and differential expression (log2 ratio, p-value) were calculated with DESeq2 in Geneious™ (2021.0.1). (Excel file)

Supplementary Table 7: Differential expression calculated for transcripts from *Hbt. salinarum* EVs normalized to intracellular levels. RNA was extracted from purified EVs (duplicates) and the respective cells (one replicate). Read mapping (90% minimum overlap identity, TPM) and differential expression (log2 ratio) performed with Geneious™ (2021.0.1). (Excel file)

Supplementary Table 8: Proteins enriched in EVs after normalization with the protein content of cell membranes. Protein content of EVs was pooled from upper and lower bands in three replicates (total of 6 EV replicates) and quantities were compared with three replicates from host cell membrane preparations. Quantity was estimated using MaxQuant (v. 1.6.10.43) and differential expression analysis (log2 fold change, adjusted p-value) was calculated with DEP (v. 1.21.0) [39]. (Excel file)

Supplementary Table 9: Proteins enriched in EVs from UV-treated cells after normalization with the protein content of respective cell membranes. Protein content of EVs from UV treated cells was pooled from upper and lower bands in three replicates (total of 6 EV replicates) and quantities were compared with three replicates from respective host cell membrane preparations. Quantity was estimated using MaxQuant

(v. 1.6.10.43) and differential expression analysis (log2 fold change, adjusted p-value) was calculated with DEP (v. 1.21.0) [39]. (Excel file)

Supplementary Table 10: Proteins identified as present in all EV samples. Protein content of EVs from untreated cells (3 replicates from upper and lower bands of density gradient each) and UV treated cells (3 replicates from upper and lower bands of density gradient each) was pooled (total of 12 EV replicates). Label-free quantities (LFQ) were calculated using MaxQuant (v. 1.6.10.43) and averaged over all replicates. Proteins were only considered present if peptide count was greater than or equal to 2 in all replicates and all replicates had a corresponding LFQ value. (Excel file)

Supplementary Table 11: Mass spectrometry peak areas and relative abundances of lipid compounds extracted from whole cells, cell membranes and EVs of *H. volcanii*. Intact polar lipids were extracted from whole cells, cell membranes and vesicles of *H. volcanii* and measured with a Q-TOF MS (Bruker Daltonics). Output data were analyzed with the manufacturer's software (DataAnalysis 4.4.2, Bruker Daltonics) and lipid compounds were identified based on retention time, fractionation pattern and exact masses and quantified via mass spectrometry peak area. (Excel file)

Supplementary Table 12: Taxonomy of Archaea identified to contain archaeal vesiculating GTPase, ArvA. 1,666 archaeal organisms out of 78,738 archaeal and bacterial genomes were identified to contain an ArvA homolog (see Methods). Taxonomy listed according to genome taxonomy database release (r207). (Excel file)

Supplementary Table 13: ArvB/C analysis. Table summarizing the number of genomes (out of 1,666) containing ArvB/C grouped with ArvA. (Excel file)

SUPPLEMENTARY REFERENCES

1. Delmas S, Duggin IG, Allers T (2013) DNA damage induces nucleoid compaction via the Mre11-Rad50 complex in the archaeon Haloferax volcanii. Mol Microbiol 87:168–179. https://doi.org/10.1111/mmi.12091
2. Manning AJ, Kuehn MJ (2011) Contribution of bacterial outer membrane vesicles to innate bacterial defense. BMC Microbiol 11:258. https://doi.org/10.1186/1471-2180-11-258
3. Martins SdT, Alves LR (2020) Extracellular Vesicles in Viral Infections: Two Sides of the Same Coin? Front Cell Infect Microbiol 10:593170. https://doi.org/10.3389/fcimb.2020.593170
4. Alarcón-Schumacher T, Naor A, Gophna U et al. (2022) Isolation of a virus causing a chronic infection in the archaeal model organism Haloferax volcanii reveals antiviral activities of a provirus. Proc Natl Acad Sci U S A 119:e2205037119. https://doi.org/10.1073/pnas.2205037119
5. Bernadac A, Gavioli M, Lazzaroni J-C et al. (1998) Escherichia coli tol-pal Mutants Form Outer Membrane Vesicles. Journal of Bacteriology 180:4872–4878
6. McBroom AJ, Johnson AP, Vemulapalli S et al. (2006) Outer membrane vesicle production by Escherichia coli is independent of membrane instability. Journal of Bacteriology 188:5385–5392. https://doi.org/10.1128/JB.00498-06
7. Abu-Qarn M, Yurist-Doutsch S, Giordano A et al. (2007) Haloferax volcanii AglB and AglD are involved in N-glycosylation of the S-layer glycoprotein and proper assembly of the surface layer. J Mol Biol 374:1224–1236. https://doi.org/10.1016/j.jmb.2007.10.042
8. Dawson KS, Freeman KH, Macalady JL (2012) Molecular characterization of core lipids from halophilic archaea grown under different salinity conditions. Organic Geochemistry 48:1–8. https://doi.org/10.1016/j.orggeochem.2012.04.003
9. Kellermann MY, Yoshinaga MY, Valentine RC et al. (2016) Important roles for membrane lipids in haloarchaeal bioenergetics. Biochim Biophys Acta 1858:2940–2956. https://doi.org/10.1016/j.bbamem.2016.08.010
10. Sprott GD, Larocque S, Cadotte N et al. (2003) Novel polar lipids of halophilic eubacterium Planococcus H8 and archaeon Haloferax volcanii. Biochim Biophys Acta 1633:179–188. https://doi.org/10.1016/j.bbalip.2003.08.001
11. Mileykovskaya E, Dowhan W (2009) Cardiolipin membrane domains in prokaryotes and eukaryotes. Biochim Biophys Acta 1788:2084–2091. https://doi.org/10.1016/j.bbamem.2009.04.003
12. Mills J, Erdmann S (2022) Isolation, Purification, and Characterization of Membrane Vesicles from Haloarchaea. Methods Mol Biol 2522:435–448. https://doi.org/10.1007/978-1-0716-2445-6_30
13. Witte A, Baranyi U, Klein R et al. (1997) Characterization of Natronobacterium magadii phage ΦCh1, a unique archaeal phage containing DNA and RNA. Mol Microbiol 23:603–616
14. Duggin IG, Aylett CHS, Walsh JC et al. (2015) CetZ tubulin-like proteins control archaeal cell shape. Nature 519:362–365. https://doi.org/10.1038/nature13983
15. Adams PP, Flores Avile C, Popitsch N et al. (2017) In vivo expression technology and 5' end mapping of the Borrelia burgdorferi transcriptome identify novel RNAs expressed during mammalian infection. Nucleic Acids Res 45:775–792. https://doi.org/10.1093/nar/gkw1180
16. Haque RU, Paradisi F, Allers T (2020) Haloferax volcanii for biotechnology applications: challenges, current state and perspectives. Appl Microbiol Biotechnol 104:1371–1382. https://doi.org/10.1007/s00253-019-10314-2
17. Gamble-Milner R Genetic analysis of the Hel308 helicase in the archaeon Haloferax volcanii
18. Dyall-Smith, Michael (2009) The Halohandbook: Protocols for haloarchaeal genetics
19. Giavalisco P, Li Y, Matthes A et al. (2011) Elemental formula annotation of polar and lipophilic metabolites using (13) C, (15) N and (34) S isotope labelling, in combination with high-resolution mass spectrometry. Plant J 68:364–376. https://doi.org/10.1111/j.1365-313X.2011.04682.x

20. Wörmer L, Lipp JS, Schröder JM et al. (2013) Application of two new LC–ESI–MS methods for improved detection of intact polar lipids (IPLs) in environmental samples. Organic Geochemistry 59:10–21. https://doi.org/10.1016/j.orggeochem.2013.03.004
21. Bale NJ, Sorokin DY, Hopmans EC et al. (2019) New Insights Into the Polar Lipid Composition of Extremely Halo(alkali)philic Euryarchaea From Hypersaline Lakes. Front Microbiol 10:377. https://doi.org/10.3389/fmicb.2019.00377
22. Yoshinaga MY, Kellermann MY, Rossel PE et al. (2011) Systematic fragmentation patterns of archaeal intact polar lipids by high-performance liquid chromatography/electrospray ionization ion-trap mass spectrometry. Rapid Commun Mass Spectrom 25:3563–3574. https://doi.org/10.1002/rcm.5251
23. Wickham H (2009) ggplot2: Elegant graphics for data analysis / by Hadley Wickham. Use R! Springer, New York, London
24. Wickham H (2011) The Split-Apply-Combine Strategy for Data Analysis. Journal of Statistical Software 40
25. Wickham H, François R, Henry L, Müller K (2022) dplyr: A Grammar of Data Manipulation
26. Raden M, Ali SM, Alkhnbashi OS et al. (2018) Freiburg RNA tools: a central online resource for RNA-focused research and teaching. Nucleic Acids Res 46:W25-W29. https://doi.org/10.1093/nar/gky329
27. Will S, Joshi T, Hofacker IL et al. (2012) LocARNA-P: accurate boundary prediction and improved detection of structural RNAs. RNA 18:900–914. https://doi.org/10.1261/rna.029041.111
28. Will S, Reiche K, Hofacker IL et al. (2007) Inferring noncoding RNA families and classes by means of genome-scale structure-based clustering. PLoS Comput Biol 3:e65. https://doi.org/10.1371/journal.pcbi.0030065
29. Jumper J, Evans R, Pritzel A et al. (2021) Highly accurate protein structure prediction with AlphaFold. Nature 596:583–589. https://doi.org/10.1038/s41586-021-03819-2
30. Holm L, Laiho A, Törönen P et al. (2023) DALI shines a light on remote homologs: One hundred discoveries. Protein Sci 32:e4519. https://doi.org/10.1002/pro.4519
31. Rinke C, Chuvochina M, Mussig AJ et al. (2021) A standardized archaeal taxonomy for the Genome Taxonomy Database. Nat Microbiol 6:946–959. https://doi.org/10.1038/s41564-021-00918-8
32. Pallen MJ, Rodriguez-R LM, Alikhan N-F (2022) Naming the unnamed: over 65,000 Candidatus names for unnamed Archaea and Bacteria in the Genome Taxonomy Database. Int J Syst Evol Microbiol 72. https://doi.org/10.1099/ijsem.0.005482
33. Evans R, O'Neill M, Pritzel A et al. (2021) Protein complex prediction with AlphaFold-Multimer
34. Hartman AL, Norais C, Badger JH et al. (2010) The complete genome sequence of Haloferax volcanii DS2, a model archaeon. PLoS One 5:e9605. https://doi.org/10.1371/journal.pone.0009605
35. Allers T, Ngo H-P, Mevarech M et al. (2004) Development of additional selectable markers for the halophilic archaeon Haloferax volcanii based on the leuB and trpA genes. Appl Environ Microbiol 70:943–953. https://doi.org/10.1128/AEM.70.2.943-953.2004
36. Wolters M, Borst A, Pfeiffer F et al. (2019) Bioinformatic and genetic characterization of three genes localized adjacent to the major replication origin of Haloferax volcanii. FEMS Microbiol Lett 366. https://doi.org/10.1093/femsle/fnz238
37. Ng WV, Kennedy SP, Mahairas GG et al. (2000) Genome sequence of Halobacterium species NRC-1. Proc Natl Acad Sci U S A 97:12176–12181. https://doi.org/10.1073/pnas.190337797
38. Erdmann S, Tschitschko B, Zhong L et al. (2017) A plasmid from an Antarctic haloarchaeon uses specialized membrane vesicles to disseminate and infect plasmid-free cells. Nat Microbiol 2:1446–1455. https://doi.org/10.1038/s41564-017-0009-2
39. Arne Smits , Wolfgang Huber (2017) DEP. Bioconductor
